# Cognitive Learning, Monitoring and Assistance of Industrial Workflows Using Egocentric Sensor Networks

**DOI:** 10.1371/journal.pone.0127769

**Published:** 2015-06-30

**Authors:** Gabriele Bleser, Dima Damen, Ardhendu Behera, Gustaf Hendeby, Katharina Mura, Markus Miezal, Andrew Gee, Nils Petersen, Gustavo Maçães, Hugo Domingues, Dominic Gorecky, Luis Almeida, Walterio Mayol-Cuevas, Andrew Calway, Anthony G. Cohn, David C. Hogg, Didier Stricker

**Affiliations:** 1 Department Augmented Vision, German Research Center for Artificial Intelligence, Kaiserslautern, Germany; 2 Department of Computer Science, Technical University of Kaiserslautern, Kaiserslautern, Germany; 3 Department of Computer Science, University of Bristol, Bristol, UK; 4 School of Computing, University of Leeds, Leeds, UK; 5 Department of Computing, Edge Hill University, Ormskirk, UK; 6 Department Sensor Informatics, Swedish Defence Research Agency, Linköping, Sweden; 7 Department of Electrical Engineering, Linköping University, Linköping, Sweden; 8 SmartFactory KL e.V., Kaiserslautern, Germany; 9 Department Computer Vision, Interaction and Graphics, Center for Computer Graphics, Guimarães, Portugal; Université Libre de Bruxelles, BELGIUM

## Abstract

Today, the workflows that are involved in industrial assembly and production activities are becoming increasingly complex. To efficiently and safely perform these workflows is demanding on the workers, in particular when it comes to infrequent or repetitive tasks. This burden on the workers can be eased by introducing smart assistance systems. This article presents a scalable concept and an integrated system demonstrator designed for this purpose. The basic idea is to learn workflows from observing multiple expert operators and then transfer the learnt workflow models to novice users. Being entirely learning-based, the proposed system can be applied to various tasks and domains. The above idea has been realized in a prototype, which combines components pushing the state of the art of hardware and software designed with interoperability in mind. The emphasis of this article is on the algorithms developed for the prototype: 1) fusion of inertial and visual sensor information from an on-body sensor network (BSN) to robustly track the user’s pose in magnetically polluted environments; 2) learning-based computer vision algorithms to map the workspace, localize the sensor with respect to the workspace and capture objects, even as they are carried; 3) domain-independent and robust workflow recovery and monitoring algorithms based on spatiotemporal pairwise relations deduced from object and user movement with respect to the scene; and 4) context-sensitive augmented reality (AR) user feedback using a head-mounted display (HMD). A distinguishing key feature of the developed algorithms is that they all operate solely on data from the on-body sensor network and that no external instrumentation is needed. The feasibility of the chosen approach for the complete action-perception-feedback loop is demonstrated on three increasingly complex datasets representing manual industrial tasks. These limited size datasets indicate and highlight the potential of the chosen technology as a combined entity as well as point out limitations of the system.

## 1 Introduction

As the complexity of workflows and manual tasks in industry and production increases, the need for smart user assistance systems increases as well. The development of such systems is currently an active research topic. The idea is to support people in interacting with an increasingly complex environment. From an industrial point of view, this type of technology can allow inexperienced workers to perform complex tasks efficiently, without relying on experts or intensive training. This saves both time and money.

### 1.1 Background

While user assistance systems based on augmented reality (AR) have appeared during the last years, they have mostly been based on hardwired content. However, in order to be genuinely assistive, such systems need to have cognitive capabilities enabling them to understand the user’s activities in relation to the underlying workflow patterns. Continuous interaction between user and system during task execution is needed to provide timely instructions and messages tailored to the current situation [1]. Moreover, for practical applications, these systems should be able to learn new workflow patterns from examples, generalize to different users, environments, and domains at the same time as they are mobile, *i.e.*, independent of external infrastructure. A successful assistance system contains three major enabling technologies:

1. **User guidance and feedback.** Studies show that AR is a good way to help users through complex tasks [2, 3]. Considerable research has been undertaken over recent years and there is evidence that the technology is beginning to be exploited in the market place and to be accepted by end users [4].
2. **Workflow recovery and analysis.** This is an area of on-going research, where most current solutions are rather domain specific, *e.g.* [5]. However, efforts are made to obtain more general solutions, *e.g.*, in the European projects SKILLS [6] and COGNITO [7], the latter of which provided the basis for the present work. A prerequisite for successful workflow recovery and monitoring is a certain degree of situational awareness.
3. **Situational awareness.** This is usually acquired by capturing the user’s interaction with the task space via sensors. Different types of external and wearable sensors have been used in the past. The granularity and methods to use depend on the demands of the workflow recovery and monitoring.

This article addresses all of the above areas. It presents advances to egocentric human activity capturing, learning, and monitoring as well as describes how these abilities have been linked to an intelligent AR user interface in the context of assistance for industrial manual workflows. Manual workflows are here assumed to be tasks that are carried out by a human operator and are made up of a temporally ordered set of atomic events. Each atomic event involves the hand manipulation of one or more objects or tools in the workspace.

**Fig 1:**
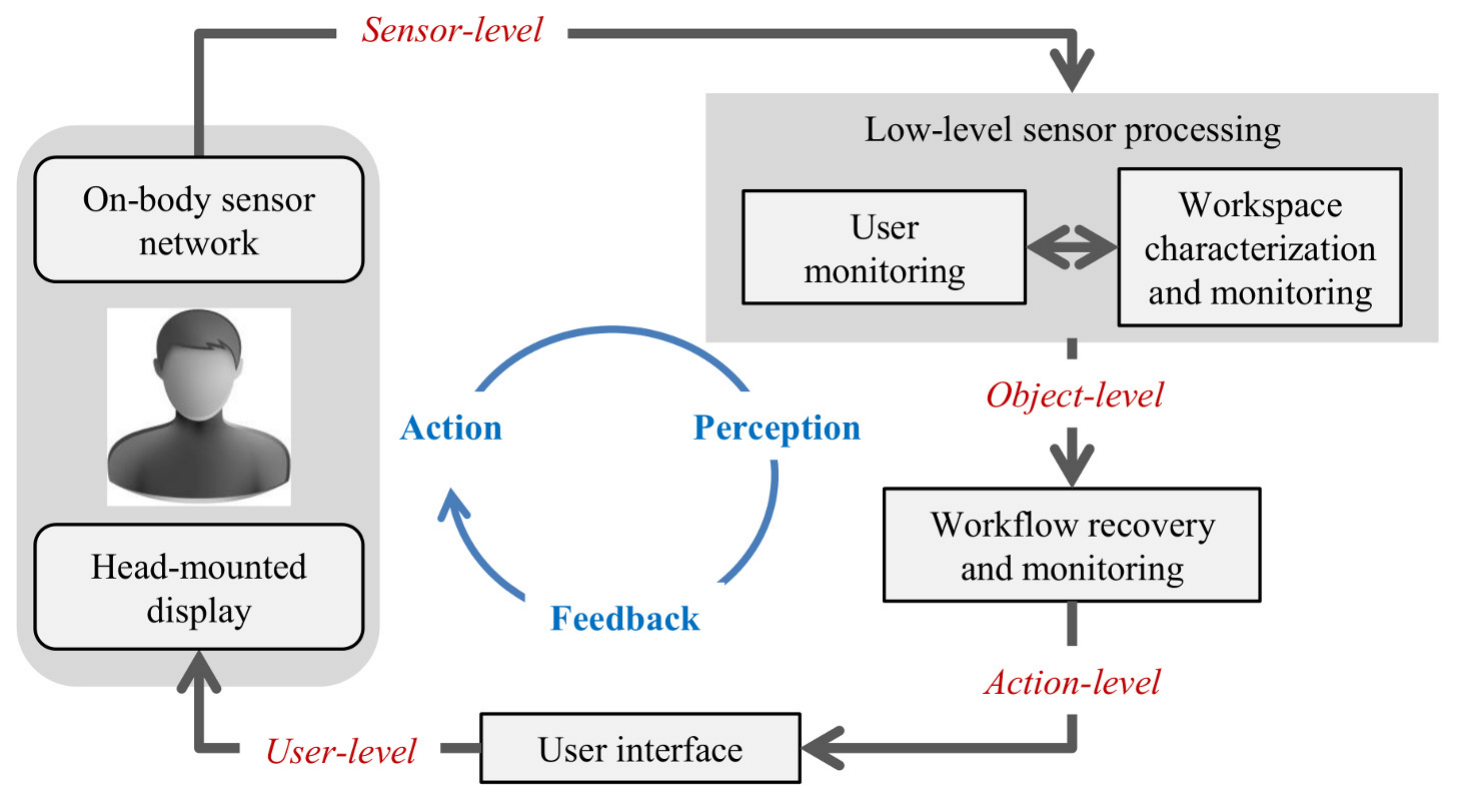
System architecture and information refinement. Gray boxes and arrows indicate system components and their interaction. The rounded rectangles illustrate the hardware platform. Starting from the on-body sensor network, the information is gradually refined from sensor- over object- to action-level information (red italics labels). Finally, it is converted into instructions and messages that are comprehensible for the user.

The assistance system suggested in this article is depicted in Fig 1. It is used in the following way: Initially, a manual workflow is learnt from demonstrations by a few experts. During the learning phase, the workflow is captured with an egocentric network of visual sensors and inertial measurement units (IMUs). More specifically, the user’s upper body motions are estimated from the wearable IMUs and an egocentric color (RGB) camera. The positions of the user’s wrists and objects in the workspace are deduced from a body-worn color and depth (RGBD) camera. In a supervised learning step, spatial and kinematic relations between pairs of objects (key objects in the workspace and the user’s upper body parts) are then learnt for each labeled atomic event. A semi-automatic authoring process allows associating the learnt workflow model with descriptive information, such as labels, text, images, video clips or 3D graphics. The learnt model and associated information then provide the basis for monitoring and assisting an inexperienced operator in executing the same task. The monitoring system recognizes the current and anticipates the most probable next event from short observation periods. It also detects deviations from the correct action. This enables tailored feedback and on-the-fly instructions based on the associated information being presented over the real world in a see-through head-mounted display (HMD).

### 1.2 Related Work

The combined system builds upon different components. The state of the art of the related research areas is described below.

#### Hardware Platforms and Motion Tracking

For the important task of collecting information about the user’s movements and activities, miniature motion and ultrasonic sensors, such as IMUs or microphones, attached to the body have been used. Previous work concerned, for instance, the recognition of physical [8], household [9, 10], and assembly task activities [11]. IMUs are also widely used for capturing detailed body motion [12, 13] without relying on external infrastructure, such as cameras [14]. However, industrial environments are often heavily contaminated by magnetic disturbances which makes this technology difficult to use.

Contextual information, *i.e.*, user pose or interactions with respect to a workspace, has mostly been obtained through visual sensors. While head tracking for AR visualizations is typically based on wearable cameras [15], user-workspace and hand-object interactions have mostly been based on stationary visual sensors [16–18]. However, this limits the user mobility and the working volume.

For presenting AR overlays, the used display hardware depends on the current technological developments ranging from rigidly installed screens and monitors [1, 19] over mobile devices, such as tablets and smartphones [20, 21], to HMD s [22]. While there is an ongoing debate about the applicability of HMD s in industrial settings, such devices are nowadays becoming more ergonomic and lightweight [23], thus starting to be accepted for daily usage.

The proposed hardware platform comprises solely wearable sensors and output devices. The body sensor network (BSN) has been designed in a way to provide sufficient information about user and workspace activity and the interaction between both. To alleviate the problems caused by magnetic disturbances, inertial sensors are combined with a chest-mounted fisheye camera to compensate for heading drift instead of the commonly used magnetic sensors [24, 25]. An HMD with integrated microphone and speakers allows for multi-modal and hands-free user feedback.

#### Scene Characterization and Monitoring

Systems developed for scene characterization and monitoring can be divided into two categories: works reliant on a static setup where the user is limited to a pre-specified area equipped with sensors [16, 26–29] and works that can deal with wearable sensors according to the proposed system [30–32].

Using wearable cameras and egocentric views for scene characterization and monitoring is quite recent. The early work of Mayol-Cuevas and Murray [30] segments the hand using skin color and represents the objects using color histograms. The recent work of Fathi *et al.* recognizes hand-held objects using a head-mounted monocular camera [31]. The approach emphasizes the importance of foreground segmentation to focus on manipulated objects and uses skin color to segment hand regions. The foreground regions are estimated by fitting a fundamental matrix to dense optical flow vectors. The method is though far from being used in real-time, as the used algorithms like super-pixel segmentation, scale-invariant feature transform (SIFT) descriptors and graph cuts are not suitable for real-time performance. Similarly, the work of Sun *et al.* uses a wearable gaze-positioned camera [32]. Skin color is used to segment hands, and edges combined with CAD (computer-aided design) models are used to localize the objects. Three-dimensional models of the hand are used to identify the grip in 27 DOF (degrees of freedom), which is then combined with object positions and identities to recognize the activity. The system was tested on two objects, a cup and a milk box, and provides trajectories of these objects using off-line processing.

The proposed framework differs from previous work in its ability to perform in real-time within a wearable setup, while scaling well with the number of objects. It learns the objects and person-dependent object/tool grips in real-time, and then provides 3D trajectories for all learnt objects while the task is being performed.

#### Activity Recognition

Similary to scene characterization and monitoring, most existing approaches for activity recognition operate on a static camera setup and, hence, a third-person view [18, 33–36]. Starner and Pentland were one of the first to use an egocentric setup of wearable sensors to recognize American sign language in real-time [37]. More recently, Fathi *et al.* [38] presented a hierarchical model of daily activities by exploring the consistent appearance of objects, hands, and actions from an egocentric viewpoint. Aghazadeh *et al.* [39] extracted novel events from daily activities and Kitani *et al.* [40] identified ego-action categories from first-person views.

Recently, there is a growing interest in multi-modal activity recognition using visual, depth and/or inertial sensors [10, 11, 16, 36]. Koppula *et al.* [18] presented a method for recognizing daily activities using a static RGBD camera. The approach uses objects, object-object relationships and object-sub-activity features for inferring activities. The method was tested on the Cornell Activity Datasets (CAD-120 and CAD-60) [41]; however, object detection and tracking were done using off-line processing. Ward *et al.* [11] proposed a method to recognize wood workshop assembly activities by using on-body microphones and accelerometers. Similarly, Chavarriaga *et al.* [10] described an approach using on-body inertial sensors and accelerometers for recognizing modes of locomotion and gestures and for automatically segmenting relevant actions using different off-line classification techniques. Note that the latter methods do not provide awareness of the user activity in relation to the workspace geometry, which is required for giving AR feedback contextualized to the current workspace configuration and user movement.

Most of the above mentioned activity recognition systems classify activities after having fully observed the entire sequence, *i.e.*, *off-line*. However, this is unsuitable for recognition of an atomic-level, incomplete and/or ongoing activity, especially in the context of an assistance system. Moreover, such systems usually expect the same number of people or objects being observed over the entire activity whilst in realistic scenarios often people and objects enter/leave the scene while activity is going on. This work proposes an activity recognition technique that overcomes these drawbacks of existing approaches. The key features are: 1) *on-line* prediction from partial observation; 2) handling of a varying number of objects being observed at different times; 3) sequential modeling of high dimensional discontinuous sparse features using hidden Markov models (HMM). Furthermore, the proposed method has been designed to be robust against noise, detection and tracking errors, which are inevitable, when it comes to complex scenes and interactions.

#### AR User Interfaces for Assistance Systems

It is a well established fact that AR systems provide benefits compared to traditional assistance methods, *e.g.*, paper manuals or linear videos, in industrial settings [3, 42]. Visual information has mainly been provided to assist complex cognitive and manual tasks. Concerning the type of visual overlays, solely relying on textual information with annotations providing a locational or directional emphasis is still the state of the art. The authors of [43] argue that it is feasible to cover a large fraction of tasks with a quite limited set of 25–30 predefined 3D annotation overlays. Some more practical systems complement the AR experience with technical 2D or 3D sketches and video sequences illustrating a certain work step. This comes at the cost of the added cognitive burden as indicated by, *e.g.*, [3]. The authors of [44] propose to overlay the previously recorded video directly onto the AR workspace. Users in their study reported that the overlaid video instructions were easy to follow. Recently, [17] proposed a combination of videos and manual annotations. Another challenging task is the intuitive presentation and indication of hidden information in AR. A popular solution is to direct the user to change his viewpoint into a more adequate configuration. Several techniques have been developed for this including attention funnels [45] and 3D arrows [46]. Few systems add acoustic or haptic feedback to the visual information in cases where the user needs more detailed explanations [46, 47] or should be alarmed in hazardous situations [48].

The proposed user interface uses different modalities (visual, audible) as well as types of visual overlays (textual overlays, static and dynamic 3D annotations, attention cues, videos) and combines this with interaction possibilities via speech commands. The amount and type of information to be provided can be configured by the user.

### 1.3 Concept and Contributions

The proposed system architecture is illustrated in Fig 1. It decouples the workflow monitoring from the raw sensor information via an intermediate low-level processing layer. This layer provides object-level information, such as positions of the user’s wrists and body parts as well as key objects. The low-level layer is person-dependent (requiring calibration for each user) while the workflow monitoring is person-independent. Decoupling the higher-level analysis from the raw sensory information using a *hierarchical processing scheme* yields a system less sensitive to changes in the sensor equipment, operator characteristics, and environmental conditions. Other distinguishing features are: *mobility*, the system is entirely based on egocentric sensing; *domain-independence and transferability*, workspace monitoring and workflow recovery are entirely learning-based without making any other assumption than that people and objects are involved in the workflow; *multi-modal information fusion*, multi-modal fusion is performed on each processing level, including visual-inertial body motion estimation, workflow monitoring based on operator motions and hand-object relations, and multi-modal user feedback in terms of textual and graphical overlays as well as audio feedback and speech interaction.

The above paragraph introduces the main pillars of the proposed work. From a technical prospective, the resulting contributions can be summarized as follows:

- Design and development of an integrated system, which addresses the complete action-perception-feedback loop, linking data capture with workflow understanding and feedback.
- Design of an egocentric sensor network and display, which allows for joint user and workspace monitoring as well as feedback in a mobile way.
- Visual-inertial motion capturing using egocentric views from a chest-mounted camera for increased robustness against magnetic disturbances and registration with the vision framework.
- Learning-based computer vision framework for scene monitoring, comprising functionalities for dense scene mapping, sensor localization, multi-object recognition and tracking for hand-held textureless tools and components with person-specific grips, all based on RGBD information from a wearable sensor and in real-time.
- On-line activity monitoring model based on learnt spatiotemporal pairwise relations between user and objects, which handles inter-person variability and varying numbers of objects and is robust against noise, detection and tracking errors.
- Multi-modal, customizable user interface exploiting the cognitive capabilities for providing tailored feedback.

### 1.4 Outline and Datasets

**Fig 2:**
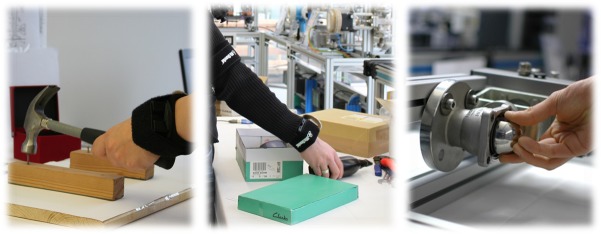
Industrially motivated test workflows. From left to right: Nails & Screws, Labeling & Packaging, Ball valve.

The article is organized as follows: Section 2 details the materials and methods developed for each building block as described above. While the focus is on the low-level sensor processing and workflow recovery and monitoring, the user interface and a formative study leading to the design of the latter are also described, along with the integrated system for workflow recovery and real-time monitoring and assistance. Section 3 presents and discusses experimental evaluation results of the system and its components. The focus is on the technical evaluation of the developed algorithms and methods. An expert evaluation of the user interface is also presented. Indicative results are provided based on three increasingly complex industrial use case scenarios (Nails & Screws, Labeling & Packaging, Ball valve) as shown in Fig 2 and Table 1. For more information, please visit [49, 50]. To the best of our knowledge, this dataset is the first of its kind to use solely on-body sensors and provide three activities of increased complexity. The article closes with a final conclusion and a discussion of limitations and future directions of the proposed technology.

**Table 1:** Test datasets and workflows. The complexity of the datasets increases from (a)–(c).

(a) Workflow: Nails & Screws	
**Summary**	Hammer 3 nails and fasten 3 screws onto a wooden piece
**Remark**	Simple operations, magnetic disturbances
**Objects**	Box, wooden baton, hammer, and screwdriver
**Atomic events**	Take/put box, take baton, take hammer, take nail/screw, hammer nail, put down hammer, take screwdriver, drive screw, put down screwdriver
**Captured data**	IMU data (3D acceleration, angular velocity, magnetic field) from 5 sensors (ColibriW from Trivisio Prototyping GmbH) (chest, upper arms, forearms) at 100 Hz, RGB images from a chest-mounted fisheye camera (IDS UI-1221LE USB color camera from IDS with VGA resolution and diagonal field of view of 180°) at 20 Hz, RGBD images from an overhead sensor (Asus Xtion Pro Live) at 30 Hz
**Collected sequences**	5 participants, 5–6 workflow executions each, 1 individual variation
**Participants**	2/3 female/male (mean age 30), list: id1, id2, id3, id4, id5 (participants are identified by their individual id, all participants were instructed prior to recording)
**Workflow designers**	Members of the COGNITO project
(b) Workflow: Labeling & Packaging	
**Summary**	Attach labels to two objects and package them within a box, then mark the box as completed using a marker pen
**Remark**	Manipulations requiring both hands, complex *N*-wise relationships between objects
**Objects**	Bottle, box, pen, tape dispenser
**Atomic events**	Take/put bottle, stick label, take/put box, remove cover, put bottle inside box, take/put cover, write address, take/put tape dispenser, seal box
**Captured data**	same as above
**Collected sequences**	5 participants, 4–5 workflow executions each, varying background clutter
**Participants**	3/2 female/male (mean age 29), list: id1, id2, id3, id5, id6
**Workflow designers**	SmartFactory engineers (initial suggestion) and members of the COGNITO project (adaptation)
(c) Workflow: Ball valve		
**Summary**	Install a new ball valve and assemble the components of a pump system
**Remark**	Manipulations requiring both hands, complex operations, many tools, shiny and small objects, magnetic disturbances
**Objects**	Ball valve, ball valve casing, electrical positioner, positioner covering, connecting pipe, screwdriver, spanner
**Atomic events**	Take/attach ball valve into base, take/attach bearing onto base, fix casing with nuts and bolts (4), take spanner, tighten nuts (2), put down spanner, take/put electrical positioner, take/fix positioner covering, take screwdriver, fasten screws of electric cover (4), put down screwdriver, attach electric positioner to actuator, fix positioner with nuts (2), take connector, attach and tighten connector, remove cap, take pipe, fix cap to pipe, attach pipe to the base
**Captured data**	IMU data from 7 sensors (chest, pelvis, head, upper arms, forearms) at 100 Hz, RGBD images from an overhead sensor at 30 Hz.
**Collected sequences**	6 participants, 4–5 workflow executions each
**Participants**	2/4 female/male (mean age 29), list: id1, id3, id5, id6, id7, id8
**Workflow designers**	SmartFactory engineers

## 2 Materials and Methods

In the following, the different system building blocks, *i.e.*, the hardware platform and the processing components as well as the integrated system are described in more detail. Note, the individuals in this manuscript have given written informed consent (as outlined in the PLOS consent form) to publish their photographs.

### 2.1 Hardware Platform

The hardware platform comprises the input and output devices of the monitoring system, *i.e.*, the components that are most tangible for the user.

**Fig 3:**
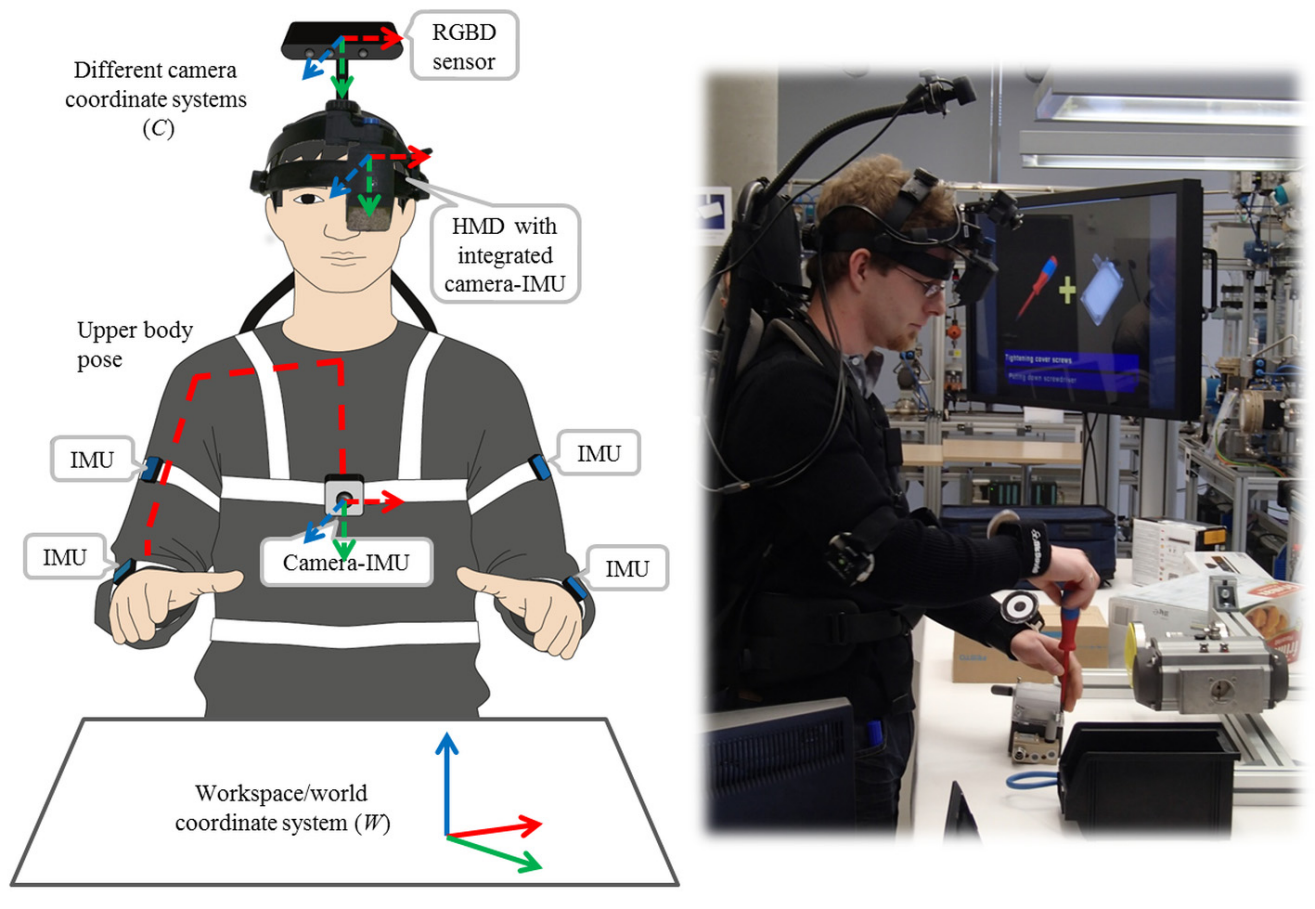
Hardware platform and sensing concept. The hardware platform consists of the BSN and the HMD, which represent the major input and output devices of the system. Left: a schematic drawing of the BSN. Right: a user wearing the setup while performing the Ball valve workflow.

**Fig 4:**
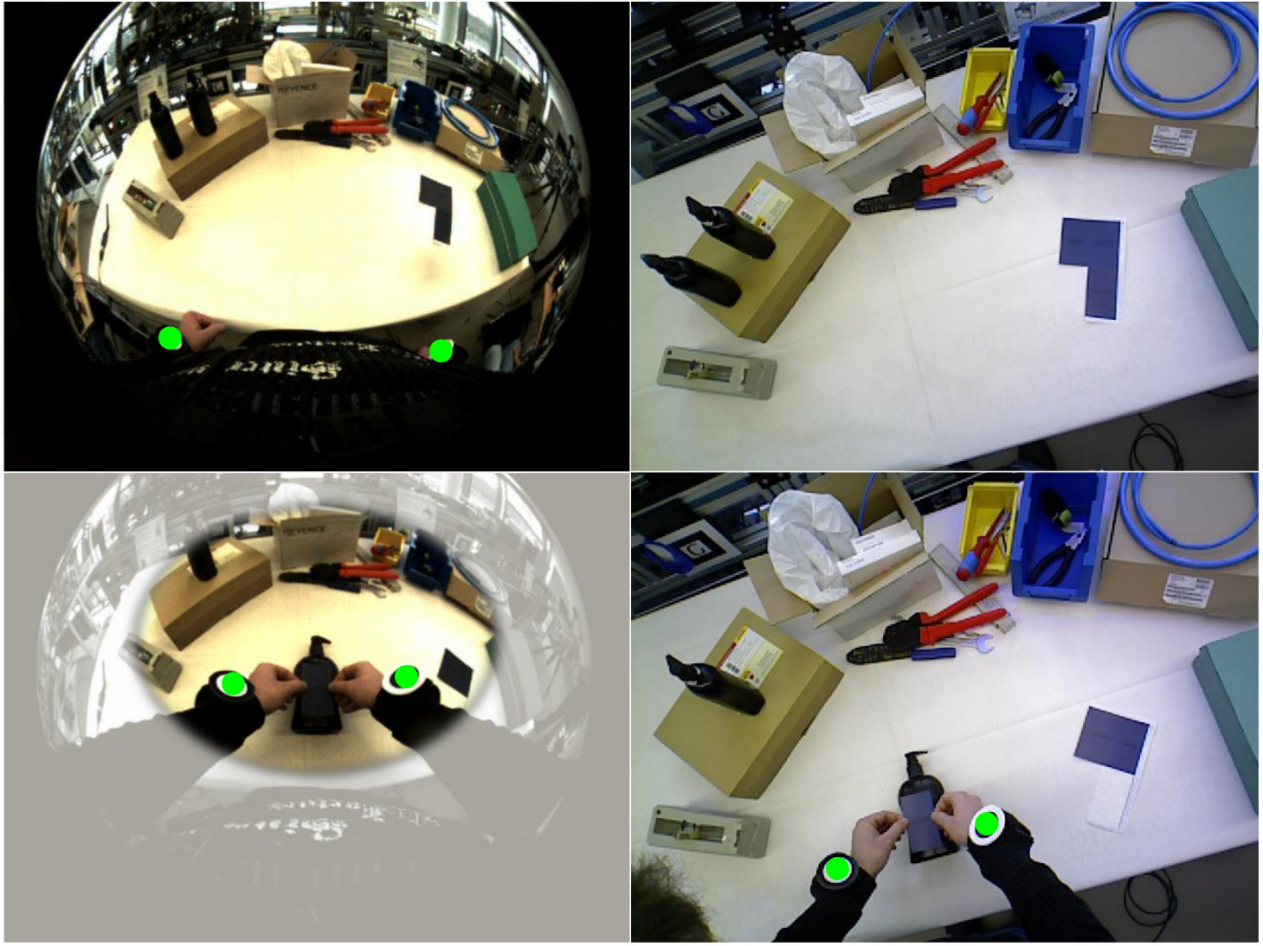
Example camera frames from chest and overhead camera. Left: the chest-mounted fisheye camera provides a good overview of the workspace where the user’s wrists (marked with green circles) are visible even if the arms are in a starting position. Right: the overhead RGBD sensor provides a narrower top view. The field of view covered with the RGBD sensor is indicated in the left lower fisheye view. Combining both views, the user activity can always be roughly tracked, while more detailed tracking is available during workspace interactions.

The BSN has been designed to provide sufficient information about user and workspace activity and the interaction between both in order to enable workflow monitoring. It comprises four wireless IMUs, two camera-IMU units and an overhead RGBD sensor. As illustrated in Fig 3, two IMUs are placed on each of the user’s arms to provide information about relative arm movements. The camera-IMU at the chest provides information about the pose of the trunk and the pose of the arms relative to the trunk. Using a wide-angle lens, this solution offers a good overview of both the workspace and the user’s own activity. A second camera-IMU integrated into the HMD enables global positioning of the head with respect to (w.r.t.) the workspace so that graphical augmentations can be rendered in the correct perspective. The overhead RGBD sensor provides a narrower and less distorted view of the workspace and delivers both color and depth information. This enables detailed detection and tracking of the user’s wrists as well as relevant objects in the workspace while being handled during task performance (*cf.* Fig 4).

The proposed user interface is based on a monocular optical see-through HMD with integrated microphone and speakers. This provides the means for hands-free user feedback in graphical and audible form, and speech interaction. In contrast to a video see-through device, which captures the reality through a camera and visualizes the video stream, the monocular optical see-through HMD still provides a free view of the workspace, which is important in the context of manual tasks.

### 2.2 User Monitoring

User monitoring tracks the user’s upper limbs, wrists and trunk using the on-body IMUs and the chest-mounted fisheye camera. The tracked joint kinematics are one input modality to workflow recovery and monitoring. In addition, the head pose is tracked relative to the workspace using the head-mounted camera-IMU. This is needed to correctly render projected 3D graphics in the HMD (*cf.* Section 2.5). While head tracking and camera-eye calibration are based on [15, 51, 52], a visual-inertial motion capturing method, which is independent of any external infrastructure and works robustly in an industrial setting, has been developed [53]. The approach is outlined in the following.

#### Body Model

**Fig 5:**
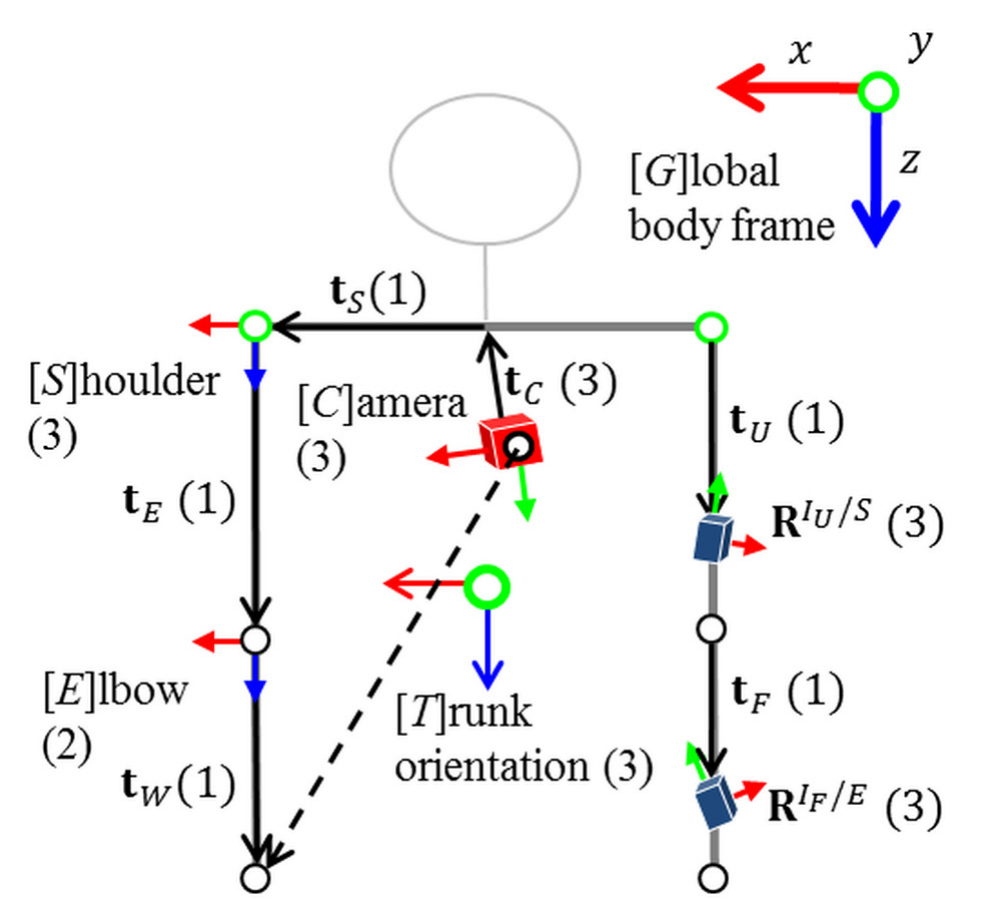
Functional upper body model with segment frames (left), technical sensor frames (right), and DOF in parenthesis. In the nominal pose, all joints are aligned with the global body-centered frame of reference, *G*, which is position-less, aligned with gravity and assumed at rest. Sensor poses are given relative to the associated joint.

The suggested tracking solution is based on a sound biomechanical model including rigid bodies (segments) and joints with dynamic anatomical constraints. Given the lengths of all the body segments, the pose is fully defined by the joint angles. The shoulder is considered a ball-and-socket joint with three DOF, whereas the elbow is modeled as a universal joint with two DOF. This reflects that the elbows cannot be bent sideways. All rotation axes are considered orthogonal. This gives a functional model with five segments (trunk, upper arms, and forearms) and anatomically motivated restricted joints (shoulders and elbows) as illustrated in Fig 5. Note, the poses of the IMUs and camera with respect to associated joints are deduced by calibration (*cf.* Section 2.6) and assumed known here.

#### Tracking Method

The joint angles and kinematics are estimated using a set of loosely coupled *extended Kalman filters* (EKFs) [54]. The visual-inertial chest unit is used to estimate the pose of the trunk analogously to the head as mentioned above. Based on this, the two IMUs attached to each arm are then used to estimate the relative poses of the arms with respect to the trunk. Each arm is handled separately in a decoupled EKF. Instead of commonly used magnetometers, wrist positions—when detected in the camera images—are utilized in order to enable tracking in the presence of severe magnetic disturbances. Here, a simple marker-based algorithm was used for wrist detection (see the markers in Figs 3 and 4). Next, the specific state-space model for the arm motion estimation is described.

#### Inertial Arm Motion Estimation

Given the calibrated biomechanical model, the arm pose is fully determined by the shoulder, **R** ^*S*/*T*^, the elbow, **R** ^*E*/*S*^, and the trunk rotation, **R** ^*T*/*G*^. See Fig 5 for an illustration of the different coordinate systems and transformations. This information is sufficient to compute the IMU orientations and positions in the global frame:

$$\begin{array}{cc} R^{I_{U}/G}=R^{I_{U}/S}R^{S/T}R^{T/G},\; & R^{I_{F}/G}=R^{I_{F}/E}R^{E/S}R^{S/T}R^{T/G} \end{array}$$ (1a)

$$\begin{array}{cc} i_{U}^{G}=R^{G/T}\left(t_{S}^{T}+R^{T/S}t_{U}^{S}\right), & i_{F}^{G}=R^{G/T}(t_{S}^{T}+R^{T/S}\left(t_{E}^{S}+R^{S/E}t_{F}^{E}\right)), \end{array}$$ (1b)

where ${\text{i}}_{A}^{B}$ is the position of IMU *A* in frame *B*. In order to obtain a minimal parametrization, also with restricted DOF, Euler angles are used to represent the joint configurations. Hence, the system state, $\text{x}={\left({\text{θ}}^{𝖳}{\dot{\text{θ}}}^{𝖳}{\overset{..}{\text{θ}}}^{𝖳}\right)}^{𝖳}$, comprises the joint angles, ***θ*** = (***θ*** ^S/T^, ***θ*** ^E/S^)^𝖳^, also represented by the rotation matrix **R** ^*A*/*B*^(***θ*** ^*A*/*B*^), their angular velocities, $\dot{\text{θ}}$, and their angular accelerations, $\overset{..}{\text{θ}}$. The joint angles are modeled as changing according to a constant angular acceleration model with white Gaussian process noise in acceleration.

The inertial measurement models relate the measured angular velocities and accelerations to the state. The accelerometers measure a combination of body acceleration, $\overset{..}{\text{i}}$, and acceleration due to gravity in the local IMU frame. Assuming that the body as a whole is at rest, the acceleration measurement models for each IMU result from differentiating (1b) w.r.t. time twice, adding acceleration due to gravity, **g** ^*G*^, and transforming the results to the local IMU frame. This model naturally handles the effects of linear accelerations resulting from arm motions relative to the trunk, given that the offsets of the IMUs w.r.t. the adjacent joints are taken into account. The gyroscope measurement models are obtained analogously by transforming $\dot{\text{θ}}$ to the local frame.

The integration of noisy and biased angular velocities results in an increasing orientation drift, which can be compensated for by the acceleration measurements only in two angles. Magnetometers are commonly used to correct the global heading direction. With the local magnetic field, **m** ^*G*^, they provide a common forward direction. The measurement equation is obtained by rotating this vector into the IMU frame and comparing it with the measured field. However, magnetometer measurements are easily disturbed by ferromagnetic materials and electromagnetic fields from machinery, *e.g.*, when handling electrical tools or tools made of iron. This results in significant estimation errors. Therefore, a drift correction based on visual wrist detections has been developed.

#### Visual Drift Correction

While the magnetometers are used as aiding sensors during calibration, the wrist positions detected in the chest camera images, **y**_*W*_, are used as aiding measurements during normal operation, since they provide information about the configuration of the arms w.r.t. the trunk. Contrary to the magnetic fields, these measurements are egocentric, *i.e.*, they are independent of external reference frames, which is a big advantage in the industrial application context. The wrist positions detected in the images are first transformed to the normalized image space, compensating for the intrinsic camera parameters. The measurement equation then results from projecting the wrist into the camera image using perspective projection:

$$\begin{array}{c} y_{W}=\frac{1}{{t_{W}^{C}}_{z}}(\begin{array}{c} {t_{W}^{C}}_{x}\\ {t_{W}^{C}}_{y} \end{array})+e_{W},\;\;t_{W}^{C}=R^{C/T}(t_{C}^{T}+t_{S}^{T}+R^{T/S}(t_{E}^{S}+R^{S/E}t_{W}^{E})), \end{array}$$ (2)

where **e**_*W*_ denotes mutually independent zero-mean Gaussian measurement noise. Here, the camera rotation, **R** ^*C*/*T*^, and position, ${\text{t}}_{C}^{T}$, w.r.t. the trunk as well as the segment lengths, **t**, belong to the set of calibration parameters. Moreover, the vector ${\text{t}}_{W}^{C}$ is indicated by the dashed arrow in Fig 5.

The above method results in an easy to setup, inertial upper body tracking system with egocentric vision support. The system provides good estimates of the upper body pose also in industrial environments, where the performance of systems based on magnetometers degrades due to magnetic disturbances (*cf.* Section 3.2). Another advantage of the system is the possibility to register the captured motions in the workspace coordinate system, *W*, rather than the body-centered frame of reference, *G*. This is possible, since the camera is registered in the trunk frame, *T*, by calibration and can be tracked visually relative to the workspace. This allows fusing all low-level processing results, *i.e.*, object-level information from the user and the scene monitoring (Section 2.3), in one consistent frame of reference before being passed to workflow recovery and monitoring. A more detailed description of the method can be found in [53].

### 2.3 Scene Characterization and Monitoring

Scene characterization focuses on modeling the workspace environment, and identifying the different tools and components relevant to the performed task. Scene monitoring then retrieves real-time labeled object-level data describing the changes in the workspace and the motion of objects relative to the workspace throughout time. Both tasks are based on information acquired from the overhead RGBD sensor (*cf.* Fig 3).

#### Proposed Framework

**Fig 6:**
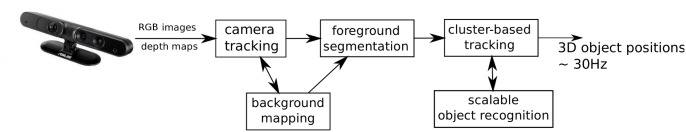
Workspace characterization and monitoring workflow. The workspace characterization and monitoring prototype allows building a map of the 3D environment, tracking an RGBD sensor using dense modeling of the workspace in terms of both depth and appearance, and segmenting outliers as foreground indicating the presence of new objects. Known objects are then recognized as well as tracked in 3D as tasks are executed.

The 3D pose of the overhead RGBD sensor is tracked in real-time and foreground objects are recognized, positioned and tracked. Object appearance and handling characteristics are learnt independently in real-time for each user. A major advantage of the proposed wearable system is that neither workspace nor objects used need to be known a priori. Fig 6 shows the different components for scene characterization and monitoring along with their interactions.

The key components can be summarized as follows:

1. Real-time technique for fusing multi-frame depth measurements captured from a moving sensor. This allows the building of dense 3D structural models of large workspaces and segmentation of foreground objects.
2. Fast (re-)localization technique for locating the RGBD sensor with respect to the workspace.
3. Scalable real-time object detection and recognition algorithm geared towards texture-minimal tools and components.
4. Tracking objects within the workspace in 3D. Coupled with the above two techniques, it allows robust recognition and tracking of tools and components within the workspace.

Details on these components are provided in the following.

#### Background Mapping, Sensor Tracking and Foreground Segmentation

Prior to task execution, a dense 3D map of the workspace is constructed by fusing data from the RGBD sensor. This sensor retrieves a temporally-synchronized image pair, containing a depth image registered to the same viewpoint as an intensity image. The first image pair is considered as the first key frame in the map. Successfully tracked frames are fused into a textured occupancy grid map in order to build a representation of the static environment. The method for background mapping uses a framework akin to the depth fusion approach of Newcombe *et al.* [55], although appearance information is incorporated. The implementation is based on the open-source KinectFusion system, which is part of the Point Cloud Library [56].

Sensor tracking estimates the sensor pose, **T** ^*W*/*C*^ ∈ 𝕊𝔼_3_, w.r.t. the common workspace coordinate system, *W*, at each new frame using the information contained in the current image and the stored map. Note, subsequently, the camera frame, *C*, denotes the coordinate frame attached to the overhead RGBD sensor, while *R* denotes the reference camera frame. The current RGBD image pair, **I** = {**I**_*D*_, **I**_*I*_}, contains a depth image, **I**_*D*_, which has been registered to the same viewpoint as the intensity image, **I**_*I*_. A similar image pair, ${\text{I}}^{\prime }=\{{\text{I}}_{D}^{\prime },{\text{I}}_{I}^{\prime }\}$, can be generated from the textured occupancy map by ray casting from a nearby, previously estimated, camera pose, **T** ^*W*/*R*^. In this section, **I** will refer to the *current* view pair and **I**′ to the *reference* view pair.

Suppose that an estimate, ${\tilde{\text{T}}}^{C/R}\in {𝕊𝔼}_{3}$, of the transformation between the current and reference view, initialized by applying a decaying constant velocity motion model to the previous estimated camera pose, is available. Then the initial estimate of the camera pose for the current view is ${\tilde{\text{T}}}^{C/W}={\tilde{\text{T}}}^{C/R}{\text{T}}^{R/W}$. Using this estimated pose, it is possible to warp the reference view images to the current view as follows:

$$\begin{array}{c} p=K{\tilde{T}}^{C/R}I_{D}^{\prime }\left(p^{\prime }\right){K^{\prime }}^{-1}p^{\prime }≔Kw\left(p^{\prime },{\tilde{T}}^{C/R}\right), \end{array}$$ (3)

where **p** and **p**′ are pixel coordinates in the current view and reference view respectively, ${\text{I}}_{D}^{\prime }({\text{p}}^{\prime })$ is the depth of pixel **p**′ in the reference depth image, and **K** and **K**′ are intrinsic camera matrices for the current view and reference view.

The optimized pose, **T** ^*C*/*R*^, is found by minimizing a nonlinear cost function formulated as a weighted combination of the intensity and depth differences. Iterative minimization of the cost function is performed using the efficient second-order approximation (ESM) [57]. Note that an extra benefit of this approach is that the same map can be used to track monocular cameras by simply adjusting the weights for intensity and depth differences in the cost function. This can be used to track the chest- and head-mounted cameras w.r.t. the workspace coordinate frame and, hence, relate the output of the user monitoring module to the shared workspace coordinate system.

With the sensor’s pose established, weighted appearance and depth differences are used to segment foreground pixels. The foreground pixels are converted into a 3D point cloud and are passed to a cluster-based tracker. Further details and evaluations on mapping, sensor-tracking and re-localization can be found in [58, 59].

#### Real-time Learning, Recognition and 3D Tracking of Objects

For each frame, the foreground segmentation is clustered into connected components based on 3D spatial proximity, with small clusters being ignored. Clusters are then assigned to trajectories maintained from the previous frame based on spatial proximity and size similarity. New trajectories are created for unassigned clusters. The tracker operates at 30 Hz. For each new trajectory, the clustered points are projected into the current frame to produce an image mask which is used to focus the object recognition algorithm.

In industrial tasks, tools and components often have little texture and adopt a wide range of 3D poses. Thus a shape-based method is proposed for object recognition. This is occlusion-tolerant, scalable and fast. The proposed method is shape-based (*i.e.*, view variant) and uses constellations of edgelets to describe each view. The sparse nature of constellations facilitates recognition in the presence of occlusion. Each constellation is described by an affine-invariant descriptor, defined in terms of the relative orientations and relative positions of the constituent edgelets.

Potentially, an exponential number of constellations is present in each view. A key feature of the method is using *fixed constellation paths*. A **path** Θ is a sequence of angles Θ = (*θ*_0_, …, *θ*_*n*−2_). From any starting edgelet, the base angle *θ*_0_ specifies the direction of a tracing vector, initially with unspecified length, relative to the orientation of the starting edgelet. If this tracing vector intersects with another edgelet in the edge map, then the edgelet is added to the constellation. This process continues until the constellation has *n* edgelets. For a traced constellation *c*_*i*_, the descriptor *f*(*c*_*i*_) = (*ϕ*_1_, …, *ϕ*_*n*−1_, *δ*_1_, …, *δ*_*n*−2_) specifies the relative orientations and distances between the consecutive edgelets in the constellation’s tuple, where *ϕ*_*i*_ = ∠(*e*_*i*_, *e*_*i*+1_) is the relative orientation of consecutive edgelets (1 ≤ *i* ≤ *n* − 1), and *δ*_*i*_ = ‖*v*_*i*+1_‖/‖*v*_*i*_‖ are the relative distances between the edgelets (1 ≤ *i* ≤ *n* − 2). The descriptor is of size 2*n* − 3, and is translation-, rotation-, and scale-invariant. By keeping a comprehensive library of descriptors for all constellations guided by one path Θ from all starting edgelets, it is sufficient to extract one constellation using the same path from the object in the test image to produce a candidate detection that is verified using the rest of the view edgelets. Further details on the real-time learning and recognition can be found in [60]. A real-time C++ implementation of the scalable textureless object detector is available at [61].

One advantage of the method is its ability to learn shape-based views in real-time. This enables learning the hand configuration of workers as they manipulate the different tools, reflecting the fact that the user grip is highly dependent on the user and the tool or component, and that many tools are heavily occluded during manipulation. This gives significant improvements in recognition performance, particularly for small tools and components.

Following recognition and tracking, timestamped information on the identities and positions of all foreground objects, including unidentified objects, are fed in real-time for workflow recovery and monitoring.

### 2.4 Workflow Recovery and Monitoring

This section addresses the modeling and reasoning methodology necessary for workflow recovery and monitoring. A workflow is defined as a temporally ordered set of procedural steps or *atomic events* for accomplishing a task in which people and tools are involved in each step of the process. The aim of the overall system is to assist operators unfamiliar with a workflow by automatically providing *on-the-fly* instructions. Therefore, the proposed framework should be able to recognize on-going events, anticipate the next possible events and recognize deviations from the correct workflow, which may lead to quality and/or health and safety problems. The workflow recovery and monitoring sub-problem consists of the following two steps:

- Workflow recovery, which provides supervised learning of statistical workflow models using object-level information provided by the scene and user monitoring module (3D positions of detected key objects and wrists as well as upper body kinematics).
- Workflow monitoring, which deduces the most likely current and next atomic event during an on-going workflow, by using the previously learnt workflow model. It receives the same object-level information as the workflow recovery step, but during live task performance.

In order to achieve the above two steps, a representation for modeling live workflow activities using *atomic events* as basic structure has been developed. This includes: 1) a sliding window based approach for modeling live activities; 2) a formalism for representing atomic events in terms of sets of qualitative spatial, temporal and functional relationships; 3) a method for recovering workflow models from geometrical and dynamical configurations of the user’s upper body parts, wrists and objects in the workspace; and 4) a method for monitoring a task in terms of a known workflow model.

**Fig 7:**
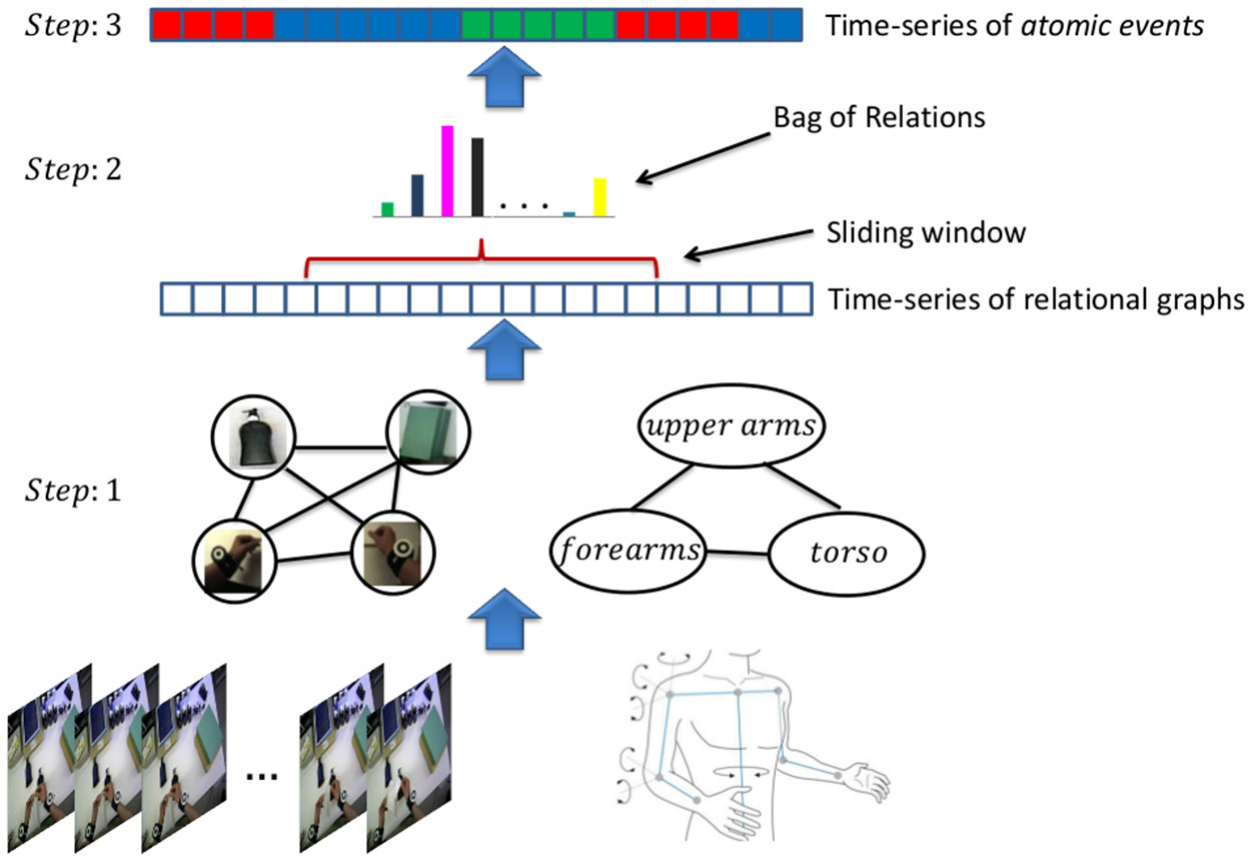
Overview of workflow recovery and monitoring framework. From bottom to top: In step 1, object-wrist and user motion relational graph structures are generated from the scene and user monitoring information. In step 2, a histogram representation of *bag-of-relations* (BoR) over a sliding window is created from the generated relational graph structures. These BoR are the feature input for the recognition of atomic events during workflow monitoring (step 3).

An overview of the proposed hierarchical framework for workflow recovery and monitoring is shown in Fig 7. *First*, atomic events are represented as interactions between objects and the user’s wrists, and between the user’s upper body parts. Interactions are here captured as spatiotemporal relations within a sliding window. A key aspect of the approach is that the spatiotemporal relations are learnt instead of being predefined manually as is common in previous work [62–64]. *Second*, atomic events are characterized by a *bag-of-relations* (BoR), which is represented as a histogram that considers the frequency of occurrences of the above-mentioned spatiotemporal relations within the sliding window. Thus, a time-series of histograms is obtained using this sliding window that moves through time. This BoR approach contrasts with logical inference from the set of relations occurring within the window. Finally, a workflow is modeled using an HMM with a conditional observation distribution over the above-mentioned time-series of histograms, whereas the HMM states are associated with the atomic events of the workflow. The observation distribution is represented by a probabilistic multi-class Support Vector Machine (SVM) which is learnt from multiple training examples in a supervised way. The whole approach, the creation of the workflow model and the real-time monitoring are described in the following paragraphs.

#### Pairwise Spatiotemporal Relations

This work is based on a representation that makes explicit spatiotemporal pairwise relationships between the user’s wrists and relevant objects observed in the workspace. Similarly, the spatiotemporal relationships between the user’s upper body parts (upper arms, forearms and torso) are established using elbow and shoulder joint kinematics. The motivation is that the former relationships are invariant to position and viewpoint, and correlate well with functional relationships between objects (*e.g.*, picking up an object with the hand involves contact between this body part and the object; hitting a nail with a hammer involves a rapid approach between hammer and nail), while the latter relationships correlate well with the start and end points of atomic events (*e.g.*, indicated by short phases of rest) and help distinguish events with characteristic motion patterns (*e.g.*, hammering, screwing).

The proposed spatiotemporal relations between objects in the workspace and the user’s wrists are based on features combining two types of information in a view-invariant fashion: 1) spatial configurations between the user’s wrists and objects in the 3D workspace coordinate system and 2) the kinematics between them over time. Although the proposed relational features are not scale-invariant, this has no significant effect in an egocentric setup, since the user only manipulates objects within his/her reach. Suppose the workspace monitoring module reports observations of objects $o_{i}^{t}$ and $o_{j}^{t}$ at time *t* at 3D positions ${\text{x}}_{i}^{t}={\left(x,y,z\right)}_{i}^{t}$ and ${\text{x}}_{j}^{t}={\left(x,y,z\right)}_{j}^{t}$ with object class types $c_{i}^{t},c_{j}^{t}\in 𝓒=\{$ *hammer*, *screwdriver*, *wrist*, *bottle*, etc.}. The spatiotemporal relation between the objects $o_{i}^{t}$ and $o_{j}^{t}$ is given by ${\text{r}}_{i,j}^{t}=(d_{i,j}^{t},\frac{{\dot{d}}_{i,j}^{t}}{d_{i,j}^{t}+\epsilon })\in {\mathbb{R}}^{2}$ [65], where $d_{i,j}^{t}=\|{\text{x}}_{i}^{t}-{\text{x}}_{j}^{t}\|$ is the Euclidean distance between objects $o_{i}^{t}$ and $o_{j}^{t}$. The kinematics, ${\dot{d}}_{i,j}^{t}$, between the objects $o_{i}^{t}$ and $o_{j}^{t}$ are captured over few frames (typically 5). The term *ϵ* is a small positive value to avoid division-by-zero errors. Using the above mentioned method, the spatiotemporal relations **r** for all possible pairs of observed objects and wrists are computed. Thereby, relations between multiple instances of the same object category are also captured.

**Fig 8:**
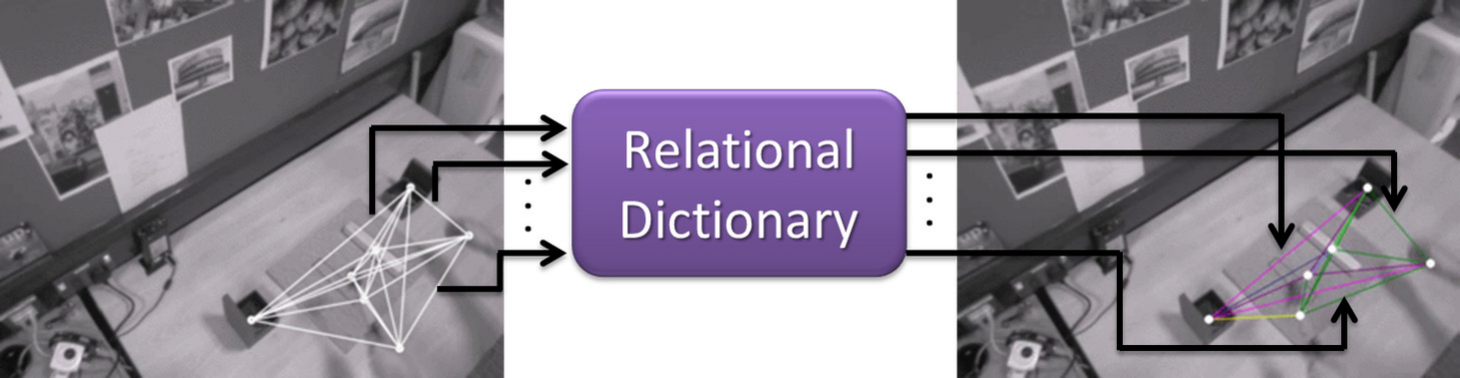
Spatiotemporal relational word. Left: spatiotemporal relations (2D vector) extracted from the observed configuration of objects and wrist 3D positions in the workspace. These relations (edges in the graph) are of the same color. Right: quantized relations using the relational dictionary corresponding to a particular object-object and object-wrist pair. Different colors are assigned to different relations (edges).

In order to overcome noise, errors, and broken tracks, which are unavoidable in vision-based tracking (*e.g.*, due to occlusions, false and/or missed detections of challenging objects, and 3D positional noise of objects and wrists), a predefined number of object-object and object-wrist discrete spatiotemporal relations is used for representing an atomic event. This implies the discretization of the 2D relation vector ${\text{r}}_{i,j}^{t}$. This is achieved by creating a relational dictionary with *K* predefined codewords per pair of objects. The dictionary per pair of objects (*o*_*i*_ and *o*_*j*_) is learnt using a *k*-means clustering algorithm. The clustering uses spatiotemporal relations **r**_*i*, *j*_, which are extracted from a training set, as input. For a given workflow, the number of key objects (∣𝓒∣) is known *a priori* and therefore the total number of unique pairs (dictionaries) is ∣𝓒∣ × (∣𝓒∣+1)/2 (including self pairing). Each relation is assigned to the closest relational word in the corresponding dictionary by using the standard Euclidean distance (see Fig 8). As a result, each spatiotemporal relation **r** is sparsely represented with ∣𝓒∣ × (∣𝓒∣+1)/2×*K* possible relational codewords [66]. The main reason for modeling individual object-object and object-wrist pairs is to capture functional relationships. For example, consider the atomic events ‘pick up hammer’ and ‘pick up screwdriver’. Both events exhibit the same spatiotemporal relations (*i.e.*, hand approaches object, gets in contact with and brings it to the workspace). The best way to distinguish these events is by using functional relationships. This is achieved by including object classes.

The relative movement of the user’s upper body parts are provided by the user monitoring module (Section 2.2) in terms of *shoulder* and *elbow* joint kinematics. These are represented as Euler angles, rates and accelerations, separately for the joints of the left and right arm. Similarly to the object-object and object-wrist relationship, the spatiotemporal relationship between the body parts is established by quantizing the individual dimensions (relative angles, rates and accelerations) via an additional dictionary with $\bar{K}$ predefined codewords. This dictionary is called the upper-body dictionary and is created similarly to the above mentioned object dictionary. Here, *shoulder* joint kinematics represent the pairwise relation between torso and upper arms. Similarly, *elbow* joint kinematics represent the pairwise relationships between upper arms and forearms. As a result the total number of unique pairs (upper-body dictionaries) is:

$$\begin{array}{ccc} & & \left|\{left\_arm,right\_arm\}|\times |\{shoulder,elbow\}\right|\\ & & \;\times \left|\left\{angles,rates,accelerations\right\}\right|\times \bar{K}=12\times \bar{K}. \end{array}$$

#### Bag-of-Relations

**Fig 9:**
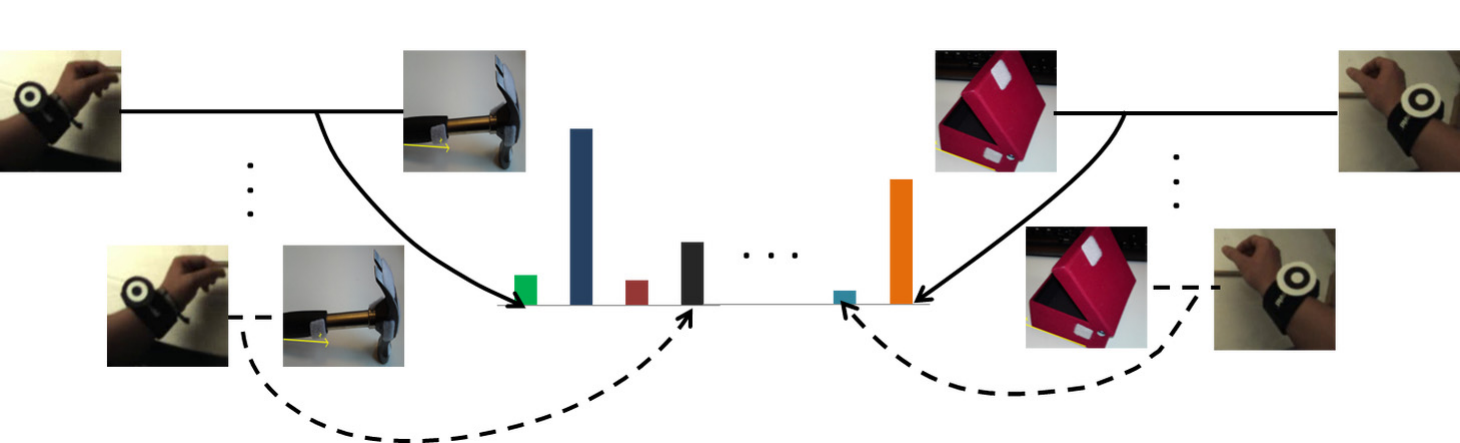
Encoding functional wrist-object relationships. Relations are accumulated separately for each pair of classes.

The workflow monitoring module receives frame-wise 3D positions of objects and the user’s wrists from the scene monitoring module (Section 2.3). Using the pairwise relations, this data is represented as an instantaneous ‘object-object-wrist’ relationship graph with nodes representing the detected objects and the wrists, and the edges depicting the spatiotemporal relationship between them (see Fig 8). The workflow monitoring module also receives frame-wise relative angles, angular rates, and angular accelerations between the user’s upper body parts (Section 2.2). This data is also represented as an instantaneous ‘upper-body’ relational graph with nodes representing the body parts and edges describing the relationships between them. These are assigned using the upper-body dictionary as presented in the above paragraph. As mentioned earlier, a sliding window is used over a fixed duration for recognizing live activities. The two relational graph structures extracted for each frame within a fixed time window (typically 1–3s) are used to summarize the on-going activities. This is done by computing ‘object-object-wrist’ (*h*_1_) and ‘upper-body’ (*h*_2_) histograms using the respective instantaneous relational graphs within the sliding window as described above. These histograms represent BoR by recording the frequency of each relation appearing in all relational graphs within the window. They are of fixed length (see Fig 9). The above two histograms are concatenated to represent a single histogram *h* = [*h*_1_, *h*_2_], which is the input feature vector for our activity recognition.

#### Workflow Model Creation

The proposed approach for workflow monitoring is model-driven. Workflow models are learnt in a supervised fashion by considering multiple training examples of workflow execution, each one processed by the low-level sensor processing modules to obtain the aforementioned object-level information. Using a sliding window approach, this information is then represented as a histogram *h* (BoR) per window according to the above paragraph. *Atomic event* labels *l* are assigned to each sliding window using voting, *i.e.*, by counting the frequency of each label (as assigned to each frame) within the window and selecting the label with the maximum votes as the winner. For the training sequences, atomic event labels are assigned to each frame through human annotation. Finally, a workflow sequence is represented as a sequence of histograms *h* computed over the sliding window. The goal is to compute the distribution of atomic events *e* for each *h*. This is achieved by training a supervised multi-class classifier using *h* with assigned label *l*, *i.e.*, by learning a discriminative function *e* = *f*(*h*). In particular, the function *f* is learnt through a probabilistic multi-class SVM [67] with *χ* ^2^ kernel. The latter has been chosen due to its better performance compared to other additive kernels such as intersection and Hellinger for histogram-based classifications [68]. The learning process uses ‘one-vs-one’ methodology.

On-line workflow monitoring, *i.e.*, tracking of a sequence of atomic events, is based on a given sequence of observed histograms using the probability distribution *P*(*e*_1_…*e*_*T*_∣*h*_1_…*h*_*T*_). In order to achieve this, the following probabilities are learnt from the training examples: 1) transition probabilities *P*(*e*_*t*_∣*e*_*t*−1_) between atomic events; 2) starting probabilities *P*(*e*_1_) for each atomic event; and 3) the distribution of atomic events given workflow activities. This establishes the temporal links between the atomic events. Moreover, these links along with the distribution *P*(*e*_*t*_∣*e*_1_…*e*_*t*−1_) provide the most likely workflow activity [66]. The result of workflow creation is a statistical workflow model that serves as input to on-line workflow monitoring.

#### Real-time Workflow Monitoring

For workflow monitoring during an assistance phase, the type of workflow is known a priori. Hence, the previously learnt workflow model is exploited for predicting the current and most likely next atomic event. This is based on the continuous stream of object-level information provided by the scene and user monitoring modules during system operation. As mentioned above, a histogram ${\bar{h}}_{t}$ is computed over a sliding window using the proposed pairwise spatiotemporal relationships based on the received data. This histogram is fed to the workflow model in order to predict the most likely current event ${\bar{e}}_{t}$ and next event ${\bar{e}}_{t+1}$ as follows:

$$\begin{array}{ccc} {\bar{e}}_{t} & = & arg max\{P\left({\bar{e}}_{t}|{\bar{h}}_{t}\right)\propto P\left({\bar{e}}_{t}\right)P({\bar{e}}_{t}|f\left({\bar{h}}_{t}\right))P\left({\bar{e}}_{t}|{\bar{e}}_{t-1}\right)\}\\ {\bar{e}}_{t+1} & = & arg max\{P\left({\bar{e}}_{t+1}|{\bar{e}}_{1},{\bar{e}}_{2},\cdots ,{\bar{e}}_{t}\right)\} \end{array}$$ (4)

The action-level information in terms of current and next event is then passed to the user interface.

### 2.5 User Interface

The user interface provides the means for editing recovered workflows (*e.g.*, labeling atomic events) and enriching them with descriptive multimedia based information through the *workflow editor* as well as assisting the user during task execution through the *AR player*. In terms of output devices, the latter uses the optical see-through HMD with integrated microphone and speakers as described in Section 2.1. The AR player is driven by the action-level information (the current position in the workflow and the next atomic event) provided by the workflow monitoring as well as object-level information necessary to render 3D graphics registered to the dynamic real world (the user’s head pose, the position of key objects in the workspace) provided by the user and scene monitoring. With this information and cognitive capabilities it is possible to provide user feedback tailored to the current workflow context, workspace configuration and user activity. In the following, the design of the AR player is described. Detailed information about the workflow editor can be found in [69].

**Fig 10:**
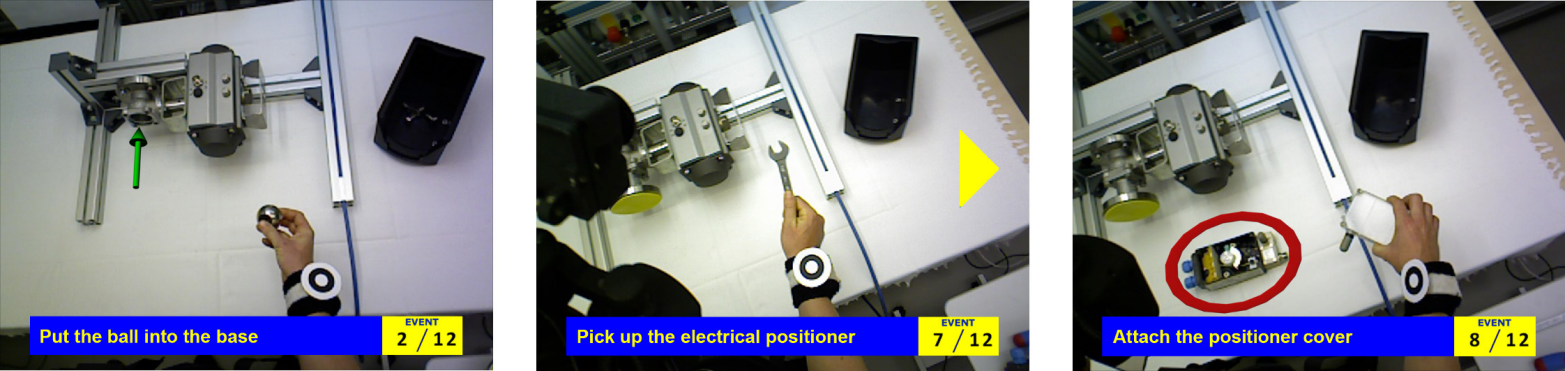
Overlays visualized in the HMD. Textual descriptions provide information on the current action and its position in the overall workflow. Relevant directions are indicated by 3D arrows. Relevant objects are highlighted by red circles (regions of interest). The yellow arrow at the right margin of the middle picture directs the attention of the user to the relevant part of the workspace, where in this case the electrical positioner is located and highlighted by a region of interest.

The AR player is the major component of the user interface. It uses AR techniques to enrich the actual workspace context with additional information at the right time, *i.e.*, the current position in the workflow and the predicted next action, and on the right locations, *e.g.*, highlighting relevant key objects, such as tools and parts, or indicating fastening points. Both textual labels and verbal descriptions are used, since, according to the multimedia effect, the combination is superior compared to either modality alone [70–73]. Additional 3D animated graphical overlays like arrows, lines, and boxes are provided, since they illustrate location and direction as well as dynamic and functional information [74, 75]. Examples are shown in Fig 10. A video presenting the AR player when being controlled via speech commands is available at [7] (Demos: Augmented Reality Display).

The AR player was developed iteratively by applying a formative evaluation approach [76]. For this, both potential users and usability experts were involved. The procedures and the final design will be described in the following.

Aiming at specific design decisions for instructions, human subjects may give hints on appropriate instructional designs, *e.g.*, the necessary content and length of verbal descriptions, the type of visualizations and additional need for support in critical operations [77, 78]. Therefore, one female and nine male subjects (mean age 26) studying engineering (six students), computer science (two students) or others (two students), were recruited from the Technical University of Kaiserslautern. They were asked to hand-design instructions for the Ball valve workflow which was unknown to all of them. For creating individual instructions the participants used a software tool called IBES [79, 80]. They were shown a video of the Ball valve task once and then asked to segment the workflow in meaningful steps, add textual descriptions, and choose graphical overlays for each sub-step.

As a result, for the Ball valve workflow, the participants annotated the directions of movement and the positions of parts by arrows and boxes, respectively. These two types of overlays were also reported as important in a previous study [74]. The results of the study served as input for creating 3D animated object categories to be augmented over the workspace through the HMD. Similarly, the segmentation into atomic events, and the textual and verbal descriptions for each event were defined for the Ball valve workflow based on input from the user study.

The prototype was then designed taking the users’ input into account. In a second iteration, four male usability experts recruited from the German Research Center for Artificial Intelligence evaluated the prototype in the context of a heuristic evaluation [81] in order to find violations to usability principles and suggest means for improvement. They were not familiar neither with the prototype nor with the details of the workflow.

The experts’ feedback was then incorporated into the user interface and applied to the Ball valve workflow. The final user interface as perceived by a user executing the task, has the following main features (*cf.* Fig 10):

- Based on the action-level information from the workflow monitoring component, the AR player indicates the current position in the overall workflow and shows a short textual instruction for the action to be performed.
- Other multimodal options like additional auditory explanations for more detailed information or images and video clips illustrating the action to be performed can be triggered via a speech command. The audio feedback prevents overloading the display with graphical information and distracting the user from the workspace.
- Alongside with unregistered overlays, the AR player also provides animated 3D graphics contextualized to the current workspace configuration. This is based on available object-level information from the scene and user monitoring and includes circles marking regions of interest (*e.g.*, the next tool or part to use) and animated 3D arrows indicating fastening locations, screwing directions, and ways of assembly.
- In order to compensate for the rather small field of view of the HMD, which does not cover the entire workspace, attention cues (flashing arrows) have been integrated to guide the user’s attention to augmented 3D annotations that are hidden in the current viewpoint (*cf.* Fig 10).

### 2.6 Integrated System

**Fig 11:**
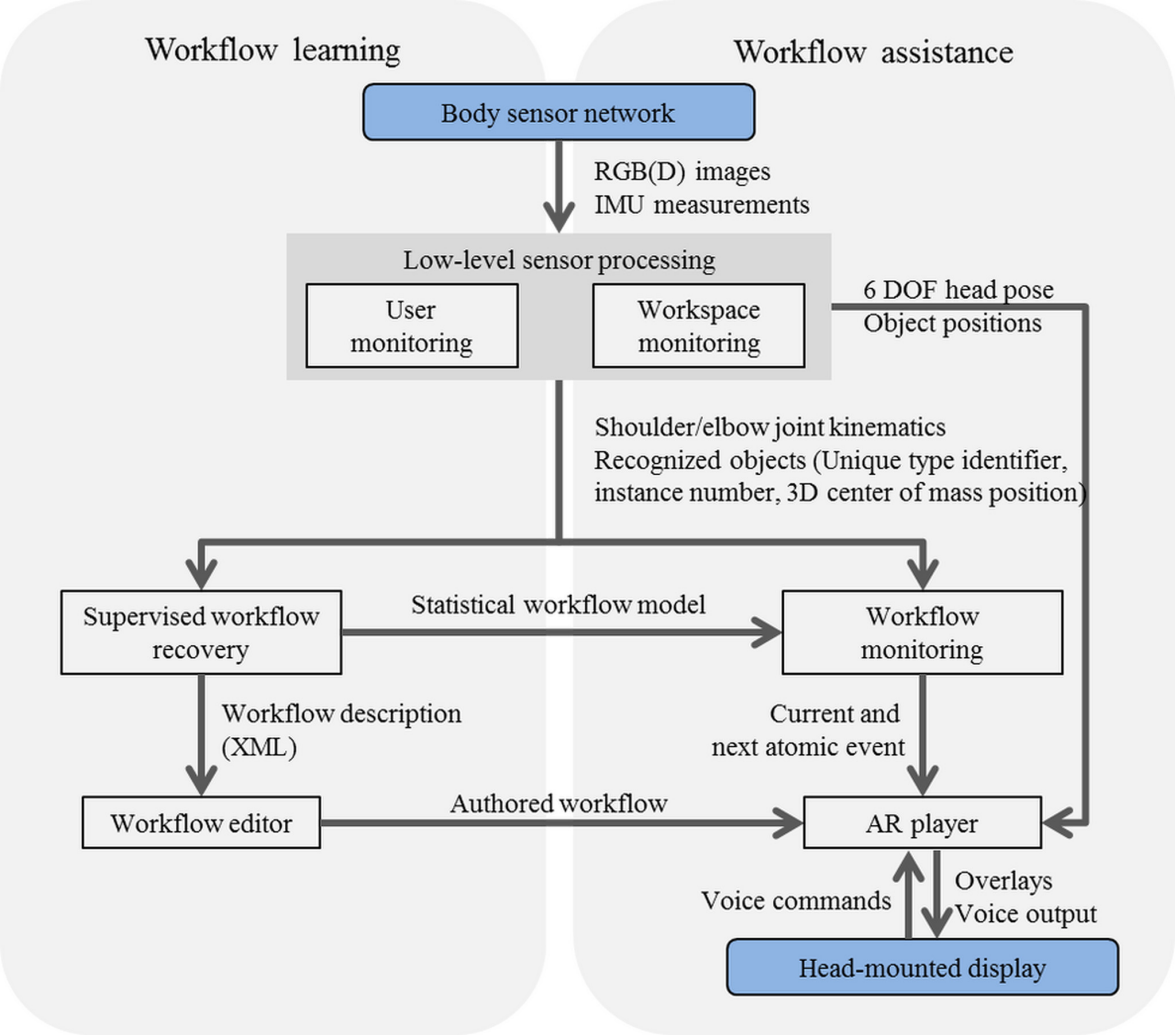
Workflow learning and online monitoring and assistance system. The left side of the diagram shows the pipeline for workflow learning. The right side shows the online monitoring and assistance system with the real-time interfaces between the building blocks. Here, the rounded rectangles mark the hardware components. BSN and low-level processing components feed into both learning and online monitoring.

After describing the methods and functionalities of each building block separately, this section is dedicated to the integrated system, its operation and its implementation. The overall working principle and the relation between workflow learning and assistance are outlined in Fig 11.

#### Hardware

For the BSN as described in Section 2.1, all sensing devices have been manufactured by the company Trivisio Prototyping GmbH apart from the real-time RGBD sensor, which is an off-the-shelf real-time structured light sensor (Asus Xtion Pro Live). The measurements from all on-body sensors are timestamped and synchronized. While the used sensors provide the required functionality for the proposed prototype, it is clear that this is not the minimal set of sensors, *e.g.*, the overhead RGBD sensor and the chest-mounted fisheye camera could be replaced by one wide-angle RGBD-IMU unit mounted on the HMD. However, as the prototype uses an off-the-shelf RGBD sensor, which requires that it is mounted at least 60 cm above the workspace, where depth information is required (the sensor was therefore attached to a backpack using an adjustable stand), and provides a limited field of view, this was not achievable. Moreover, current trends towards sensor miniaturization, low-energy wireless transmission, smart garment and high-performance wearable computing also suggest that it is feasible to use such a BSN in a real industrial environment (*cf.* [82] for ongoing work in this area).

The HMD version has been selected based on the results of a pre-study as reported in [83]. It has a diagonal field of view of 29°, SVGA resolution and has been provided by the company Trivisio Prototyping GmbH.

#### Pre-Operation

Three per-operation stages are required before workflow execution. This applies to both, the workflow recovery and the assistance phase. The first is the sensor-body calibration procedure. The second is building a map of the workspace. The third is the learning of hand-held tools and objects in the workspace in a person-specific workflow-independent manner.

Assuming that the operator wears the BSN, the local coordinate frames associated with the IMUs and the camera need to be aligned with the anatomical reference frames to provide consistent motion tracking (*cf.* Fig 5). For this, an easy-to-perform calibration procedure has been developed. It requires the user to assume two static poses and then to move the arms slowly in front of the camera a few seconds. The method automatically computes the sought-after rotations and offsets based on the data captured during the calibration and the height of the user.

Mapping the unknown workspace is based on the overhead RGBD sensor and the method outlined in Section 2.3. The map is built in real-time as the operator approaches the working space and surveys the area by rotating the body slowly to the right and left. The system assumes that a leveled pattern with known dimensions is used in the first frame to establish a common world coordinate system (*z*-axis direction and scale in accordance with global frame *G*) between the RGBD sensor and the chest- and head-mounted camera-IMU units. This common workspace coordinate frame, denoted *W*, is shared between all system components as illustrated in Fig 3.

To accommodate for different user grips, and potentially different tool shapes in different workspaces, relevant objects are learnt prior to operation. The operator is asked to grab each object/tool around the workspace, one at at time, and the system will learn the object’s shape and the user’s grip in real-time. Notice that this is workflow-independent and is required once per operator. For the three workflow scenarios, during both mapping and learning, the operator was given verbal instructions, first to rotate the body and then to manipulate the objects in turn. Each of these processes takes a few minutes.

#### Real-time System

The different interfaces and communication channels of the on-line workflow monitoring and assistance system are outlined in Fig 11 (right). Note, the diagram represents a realization of the system architecture presented in Fig 1. All processing components are implemented in a self-contained way using TCP/IP interfaces for data communication based on customized protocols. The AR player is based on the open source rendering engine Irrlicht [84]. For the experiments, the real-time monitoring and assistance system was distributed over multiple machines, which were connected to a local area network. To efficiently transfer considerable amounts of data, in particular between the two low-level processing components, and visualizing intermediate results, the well-known robot operating system (ROS) platform was used [85]. This platform provides, for instance, hardware abstraction, device drivers, visualizers as well as message-passing and package management functionality.

**Fig 12:**
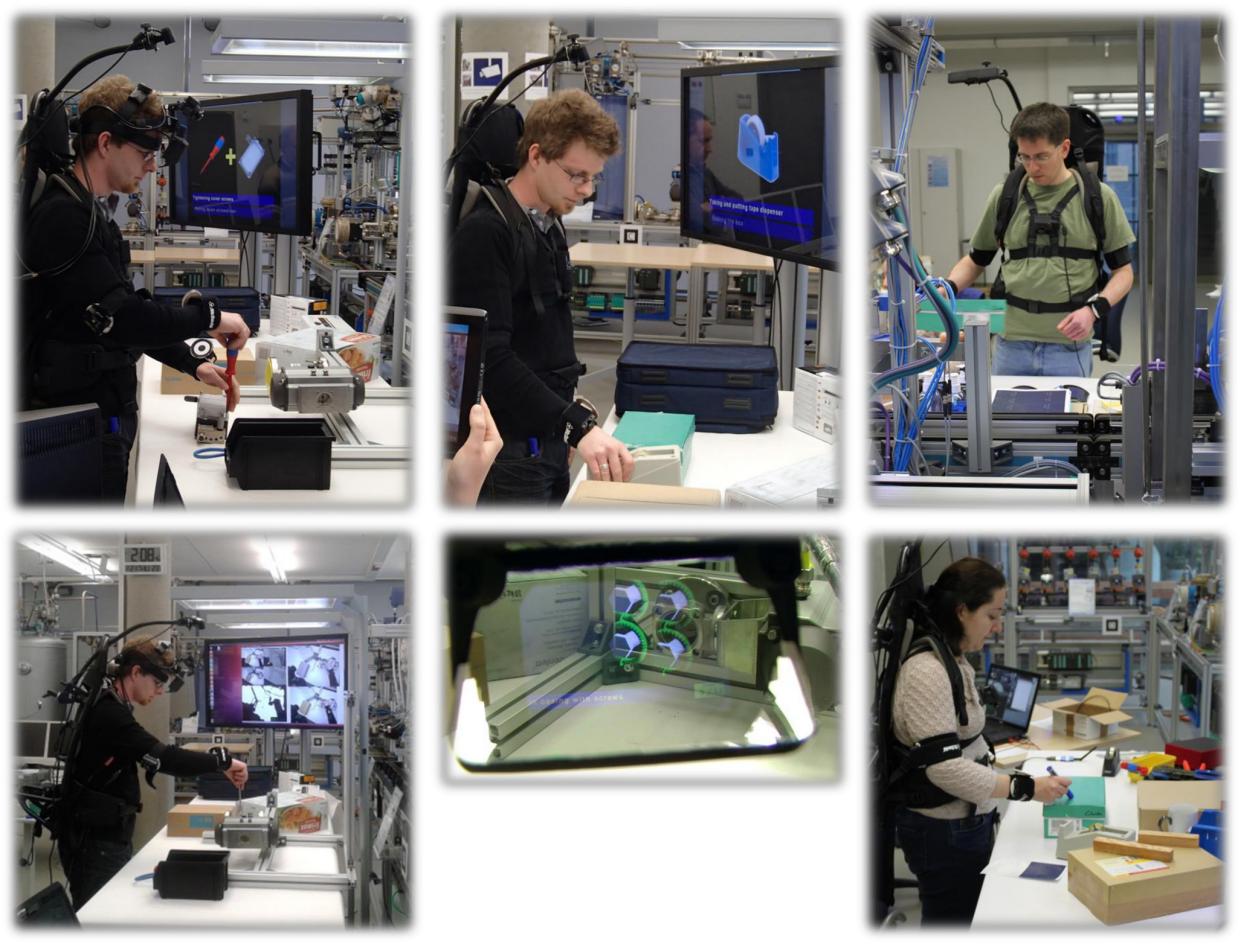
Images of the live monitoring system.

Some pictures showing the running live system with different operators and applied to different workflows are given in Fig 12. In the figure, the images on the left side show an operator wearing the complete BSN and HMD. The person is assisted in performing the Ball valve workflow. The big screen shows graphical information, such as pictures and labels, concerning the current step in the workflow (upper left) or results of the underlying workspace monitoring system (lower left). The operator himself receives textual hints and animated in-situ graphics rendered in the HMD (bottom middle). The upper middle image shows the same operator executing the Labeling & Packaging workflow while being monitored. The images on the right side show different operators performing the Labeling & Packaging workflow for workflow learning.

## 3 Results and Discussion

Three increasingly complex test datasets based on industrially motivated workflows have been captured for developing and evaluating the proposed system. The number of participants in the three activities was five, five and six, respectively, which is comparable to the standard Cornell Activity Dataset (CAD) [41]. Importantly, the activities we introduce are significantly longer in duration (2–5 minutes per sequence as opposed to 17.5 seconds for the CAD dataset, both recorded at 30 Hz). During the data capture, the preparation step was executed according to Section 2.6. The datasets are illustrated in Fig 2 and described in more detail in Table 1. This section provides quantitative and qualitative evaluation of the proposed monitoring system. Admittedly, despite the increased complexity, and having a comparable number of participants to available datasets, the number of participants and variations make it difficult to guarantee that the results could be reproduced with different participants and variations of the scenarios. However, we believe that the proposed method and the provided preliminary results would generalize well to such variations. These preliminary results demonstrate the feasibility of the system and its components as well as limitations and areas for future research. First, the overall recognition capability of the integrated framework, in terms of the prediction accuracy of atomic events, is presented in Section 3.1. The low-level sensor processing components are then evaluated explicitly in Section 3.2. Finally, a qualitative summative evaluation of the user interface is presented in Section 3.3.

### 3.1 Technical Evaluation of Workflow Recovery and Monitoring

In order to enable supervised workflow learning and evaluation of the workflow monitoring performance, all captured data sequences were manually annotated by assigning atomic event labels at camera framerate. A total of 9 atomic events was assigned to Nails & Screws and Labeling & Packaging, while Ball valve is represented by 19 atomic events (*cf.* Table 1).

**Fig 13:**
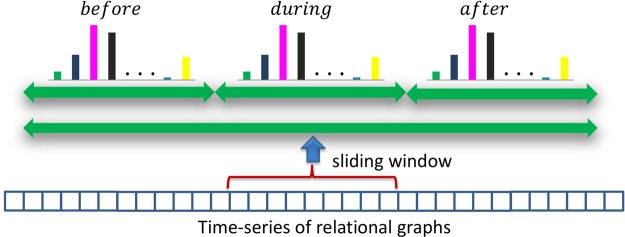
BoR calculation with subdivided sliding windows. In order to capture the temporal relations of *before*, *during* and *after*, the histogram representing the BoR is subdivided into three histograms representing the respective temporal relations.

As previously summarized, workflow recovery and monitoring receives synchronized object-level information comprising observed objects and their 3D positions as well as relative movement of the user’s upper-body parts from scene and user monitoring. For the three test datasets, this information was then characterized as histograms representing the BoR as described in Section 2.4. Histograms were calculated over a sliding window of three seconds with 50% overlap. Note that the major reason for using BoR over a sliding window is to add robustness to object detection errors, *e.g.*, due to incorrectly assigned labels. The sliding window was further divided into three equal sub-windows in order to encode the temporal relations of *before*, *during* and *after* (see Fig 13). The BoR structure is then a concatenation of the corresponding sub-histograms. Adding this temporal separation results in a significant performance improvement as further detailed in [66]. Event labels were assigned to histograms representing sliding windows via frame-based voting.

#### Evaluation Methods

*Leave-one-subject-out* was used for evaluation. In this, the data sequences captured from one subject (5–6 examples) are used for validating the model created from all other data sequences (25–30 examples from 5–6 participants). This is based on the assumption that the system is trained from workflow demonstrations of different experts, while a naïve worker is assisted afterwards. Accordingly, for each of the industrial workflows, a dedicated workflow model, atomic event transitions and prior probabilities were learnt from the training sequences as described in Section 2.4. These models were then evaluated on the unseen workflow examples considering three settings: (1) *window-classification*: atomic event predictions were optimized by assuming each sliding window was an independent sample; (2) *on-line*: predictions were boosted over a sliding window until the current time step, *i.e.*, by considering the history of events; (3) *off-line*: event predictions were optimized over the entire sequence. The *on-line* approach is required for live workflow monitoring. The evaluation was based on the classification accuracy of sliding windows. Wrongly recognized atomic events are counted as incorrect detections.

In order to compare the proposed approach with the state of the art, a baseline *bag-of-features* (BoF) method was implemented based on STIP (Space-Time Interest Points) descriptors as described in [86]. A visual vocabulary was generated by randomly sampling a subset of 100000 STIP descriptors from the training image sequences using *K*-means clustering to generate a dictionary of 4000 visual words. Descriptors were assigned to their closest visual word using the Euclidean distance and a histogram of visual word occurrences was computed over each sliding window as described above. Note that the extraction of STIP features is an off-line process, which requires a sequence of frames. It is therefore more suitable for applications requiring recognition after completion of an event. Results for both approaches were achieved using a *χ* ^2^ kernel and multi-class classifications using an SVM classifier. The histogram normalization was fixed to the *L*1-norm (both proposed BoR and the state-of-the-art method) and the parameters of the SVM classifier were optimized using 10-fold cross-validations on the training examples [66].

#### Results and Discussion

The *window-classification*, *on-line* and *off-line* performances of the *leave-one-subject-out* experiments on the three datasets are presented in Tables 2–4, respectively. In the following, *Object* refers to using only object-level information deduced from the overhead RGBD sensor (*i.e.*, object-object and object-wrist relations), *IMU* refers to using only object-level information deduced from the IMUs and chest-mounted camera (*i.e.*, upper-body kinematics), while *STIP* refers to using the above described BoF approach.

**Table 2:** Window-classification workflow monitoring performance [%]. The sliding window based average performance comparison (including standard deviations) using only SVM is shown for the *leave-one-subject-out* experiment. The proposed *bag-of-relations* (BoR), in different variants, is compared with the state-of-the-art image-based STIP (Space-Time Interest Points) features.

**Dataset**	**leave-one-subject-out**	**BoR (object-object-wrist)**	**BoR (upper-body IMUs)**	**STIP**	**BoR (object-object-wrist + upper-body IMUs)**	**BoR (object-object-wrist) + STIP**	**BoR (upper-body IMUs) + STIP**	**BoR (object-object-wrist + upper-body IMUs) + STIP**
Nails & Screws	**subject 1**	58.5	53.2	65.4	71.4	68.0	65.6	74.0
	**subject 2**	65.8	62.6	70.8	72.0	78.5	71.8	78.5
	**subject 3**	68.3	53.4	72.4	70.2	74.5	68.0	74.8
	***subject 4***	*51.5*	*10.6*	*10.1*	*30.1*	*41.5*	*12.1*	*27.5*
	**subject 5**	66.1	64.8	66.4	77.3	75.1	75.0	83.8
	**average ± std**	**62.0 ± 6.2**	**48.9 ± 19.7**	**57.0 ± 23.6**	**64.2 ± 17.2**	**67.5 ± 13.4**	**58.5 ± 23.4**	**67.7 ± 20.4**
Labeling & Packaging	**subject 1**	52.1	39.9	43.4	58.7	64.0	52.0	61.8
	**subject 2**	55.0	50.7	62.7	65.7	63.8	61.6	69.0
	**subject 3**	58.7	48.7	62.6	75.8	72.5	67.6	79.1
	**subject 4**	70.1	56.7	61.1	82.4	83.1	69.0	87.4
	**subject 5**	49.7	47.8	61.9	67.4	67.2	57.3	72.0
	**average ± std**	**57.1 ± 7.1**	**48.8 ± 5.4**	**58.3 ± 7.5**	**70.0 ± 8.3**	**70.1 ± 7.2**	**61.5 ± 6.4**	**73.9 ± 8.8**
Ball Valve	**subject 1**	50.4	34.6	76.0	58.8	75.8	63.1	67.1
	**subject 2**	57.1	37.4	79.0	66.2	74.4	73.0	78.8
	**subject 3**	37.8	37.2	52.4	49.5	51.3	54.1	55.3
	**subject 4**	47.9	23.7	57.4	51.8	60.5	56.0	63.1
	**subject 5**	67.3	43.7	77.3	72.7	77.0	68.1	80.2
	**subject 6**	61.0	44.0	70.7	64.9	74.3	70.1	76.6
	**average ± std**	**53.6 ± 9.6**	**36.8 ± 6.8**	**68.8 ± 10.3**	**60.6 ± 8.1**	**68.9 ± 9.6**	**64.1 ± 7.0**	**70.2 ± 9.1**

**Table 3:** On-line workflow monitoring performance [%]. The sliding window based average *on-line* performance comparison (including standard deviations) is shown for the *leave-one-subject-out* experiment. The proposed *bag-of-relations* (BoR), in different variants, is compared with the state-of-the-art image-based STIP (Space-Time Interest Points) features.

**Dataset**	**leave-one-subject-out**	**BoR (object-object-wrist)**	**BoR (upper-body IMUs)**	**STIP**	**BoR (object-object-wrist + upper-body IMUs)**	**BoR (object-object-wrist) + STIP**	**BoR (upper-body IMUs) + STIP**	**BoR (object-object-wrist + upper-body IMUs) + STIP**
Nails & Screws	**subject 1**	52.5	60.6	66.3	64.0	60.6	69.0	65.6
	**subject 2**	69.4	68.2	73.8	76.5	79.1	73.2	81.9
	**subject 3**	71.3	60.2	82.1	66.4	75.6	64.8	70.5
	***subject 4***	*52.5*	*13.2*	*7.4*	*40.2*	*48.5*	*12.0*	*40.2*
	**subject 5**	70.5	76.5	80.0	85.1	76.6	86.3	86.0
	**average ± std**	**63.2 ± 8.8**	**55.7 ± 22.1**	**61.9 ± 27.8**	**66.4 ± 15.2**	**68.1 ± 11.7**	**61.0 ± 25.6**	**68.8 ± 16.1**
Labeling & Packaging	**subject 1**	56.3	42.4	44.3	62.7	69.6	50.4	68.8
	**subject 2**	65.3	58.5	67.7	69.0	68.3	68.8	70.3
	**subject 3**	58.2	52.3	75.3	77.0	72.9	72.9	77.6
	**subject 4**	61.9	65.1	56.9	85.1	81.6	64.4	85.4
	**subject 5**	55.3	58.4	64.7	71.3	71.1	64.9	74.2
	**average ± std**	**59.4 ± 3.7**	**55.3 ± 7.6**	**61.8 ± 10.5**	**73.0 ± 7.6**	**72.7 ± 4.7**	**64.3 ± 7.6**	**75.3 ± 5.9**
Ball Valve	**subject 1**	62.0	39.9	80.6	67.9	85.2	70.9	73.1
	**subject 2**	80.4	55.2	85.7	84.8	88.0	87.5	90.5
	**subject 3**	50.3	40.8	64.2	64.7	58.6	55.5	67.2
	**subject 4**	65.6	34.1	70.4	66.2	75.3	70.9	76.2
	**subject 5**	78.9	60.9	87.8	84.9	86.2	85.7	87.5
	**subject 6**	78.1	50.7	85.1	82.5	86.8	78.8	87.8
	**average ± std**	**69.2 ± 11.0**	**46.9 ± 9.4**	**79.0 ± 8.7**	**75.2 ± 9.0**	**80.0 ± 10.5**	**74.9 ± 10.8**	**80.4 ± 8.7**

**Table 4:** Off-line workflow monitoring performance [%]. The sliding window based average *off-line* performance comparison (including standard deviations) is shown for the *leave-one-subject-out* experiment. The proposed *bag-of-relations* (BoR), in different variants, is compared with the state-of-the-art image-based STIP (Space-Time Interest Points) features.

**Dataset**	**leave-one-subject-out**	**BoR (object-object-wrist)**	**BoR (upper-body IMUs)**	**STIP**	**BoR (object-object-wrist + upper-body IMUs)**	**BoR (object-object-wrist) + STIP**	**BoR (upper-body IMUs) + STIP**	**BoR (object-object-wrist + upper-body IMUs) + STIP**
Nails & Screws	**subject 1**	58.9	64.7	72.6	73.0	68.3	75.4	77.3
	**subject 2**	70.0	71.8	75.3	75.0	79.1	74.4	81.7
	**subject 3**	70.7	57.5	82.7	68.8	73.7	66.1	73.4
	***subject 4***	*56.1*	*14.0*	*6.3*	*39.0*	*47.4*	*13.0*	*37.1*
	**subject 5**	75.5	77.0	84.1	85.5	82.6	86.3	90.0
	**average ± std**	**66.2 ± 7.4**	**57.0 ± 22.5**	**64.2 ± 29.3**	**68.3 ± 15.6**	**70.2 ± 12.4**	**63.0 ± 25.8**	**71.9 ± 18.3**
Labeling & Packaging	**subject 1**	61.8	44.9	47.7	66.8	77.1	61.2	73.5
	**subject 2**	70.5	67.5	74.9	72.9	72.1	75.8	78.6
	**subject 3**	70.3	60.8	82.7	85.8	80.1	82.5	87.1
	**subject 4**	75.9	74.7	62.1	89.1	87.0	75.1	90.0
	**subject 5**	57.9	63.7	74.8	79.6	79.8	65.2	81.6
	**average ± std**	**67.3 ± 6.5**	**62.3 ± 9.9**	**68.5 ± 12.3**	**78.8 ± 8.2**	**79.2 ± 4.9**	**71.9 ± 7.7**	**82.2 ± 5.9**
Ball Valve	**subject 1**	62.1	42.1	91.3	71.5	88.2	76.0	74.1
	**subject 2**	81.0	62.0	90.0	85.7	89.7	91.3	92.9
	**subject 3**	52.6	47.2	74.8	69.7	63.4	55.1	73.6
	**subject 4**	69.6	34.5	71.4	66.5	77.8	69.1	78.8
	**subject 5**	83.4	61.7	89.8	89.5	91.0	90.2	89.6
	**subject 6**	85.2	55.2	88.7	88.3	90.7	88.5	91.8
	**average ± std**	**72.3 ± 12.0**	**50.4 ± 10.1**	**84.3 ± 8.0**	**78.6 ± 9.5**	**83.5 ± 10.0**	**78.4 ± 13.2**	**83.5 ± 8.2**

Note that the high standard deviations only permit preliminary conclusions to be drawn, from which we wish to highlight a few. As expected, the *off-line* (Table 4) performance is better than the *on-line* performance (Table 3). This is due to the Viterbi algorithm [87] observing the complete sequence, rather than basing the prediction on partial observations, *i.e.*, from the beginning to the current time step. Moreover, the *on-line* performance is better than the *window-classification* performance (Table 2), where each histogram representing a window is considered as a standalone feature (more partial). In all experiments, the performances using vision based information alone (*Object* and *STIP*, columns 3 and 5 in the tables) is better than using IMUs alone (column 4 in the tables). This indicates that the vision based approaches are superior at distinguishing object manipulations; *e.g.*, ‘pick up hammer’ and ‘pick up screwdriver’ are two different events, which often exhibit similar kinematics but involve different object categories. However, by combining the different modalities, the prediction performance becomes significantly higher than when using a single modality alone. This holds in particular for the Nails & Screws and the Labeling & Packaging dataset. This shows the benefits of the multimodal approach. While hand-object interaction information provides discrimination for the event labels, worker kinematics information helps detecting the start and end of atomic events and increases discrimination of events with characteristic motion patterns.

Furthermore, the results show that the proposed BoR approach (*Object IMU*, column 6 in the tables) on average performs better than the baseline approach (*STIP*, column 5 in the tables), except for the Ball valve dataset. This can be explained by two reasons: First, though Ball valve is more complex in terms of number of objects and atomic events (19 events) compared to Nails & Screws and Labeling & Packaging (9 events), the former contains less variations in terms of environment (background), viewpoint and user manipulation. Hence, the STIP feature based approach, which is variant with respect to these changes, still provides very good results. Second, Ball valve contains complex, glossy and small objects, which are very challenging for the scene monitoring module resulting in a higher percentage of wrong or missed detections; *e.g.*, the ball valve is often not detected. Note that a significant performance gain can be achieved by combining the approach based on the low-level visual STIP features with the proposed BoR approach, which includes semantically meaningful information (*Object IMU STIP*, last column in the tables). This again shows the benefits of multimodal fusion.

Another important observation is the performance of *subject 4* in the Nails & Screws dataset. While this particular subject is left-handed, the workflow model in the *leave-one-subject-out* experiment was learnt using the workflow executions from right-handed subjects only. As shown in the tables, the performance for this particular subject is very low for all configurations except for the proposed BoR approach using object-object and object-wrist relations, which is robust against the handedness.

In combination, the above observations show: One of the main benefits of the proposed approach is its by design invariance with respect to varying user and workspace conditions. This is thanks to the intermediate low-level processing layer providing object-level information with reduced dependence on user and environment conditions. Based on this decoupling of workflow analysis and raw sensory information, it is also easily possible to use other existing detection and tracking modules. At the same time, this concept also introduces a dependence on the low-level sensor processing performance. While the proposed workflow monitoring approach has been designed to be robust against noises and errors in low-level sensor processing, an improved performance of the latter would, however, clearly be beneficial. Section 3.2 provides individual evaluation of the low-level sensor processing components.

**Fig 14:**
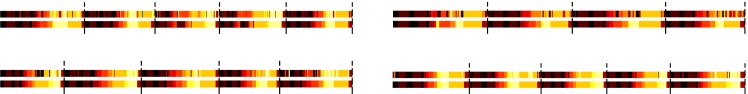
Predicted atomic events vs. ground-truth for the *leave-one-subject-out* evaluation of the Labeling & Packaging dataset. Each figure represents the predictions of the atomic events in sequences belonging to the left-out subject (left to right, top to bottom: subject 1,2,3,4). The bottom bars show the ground truth and the top bars show the prediction. The vertical lines separate two consecutive workflow sequences. Different colors indicate different atomic events.

**Fig 15:**
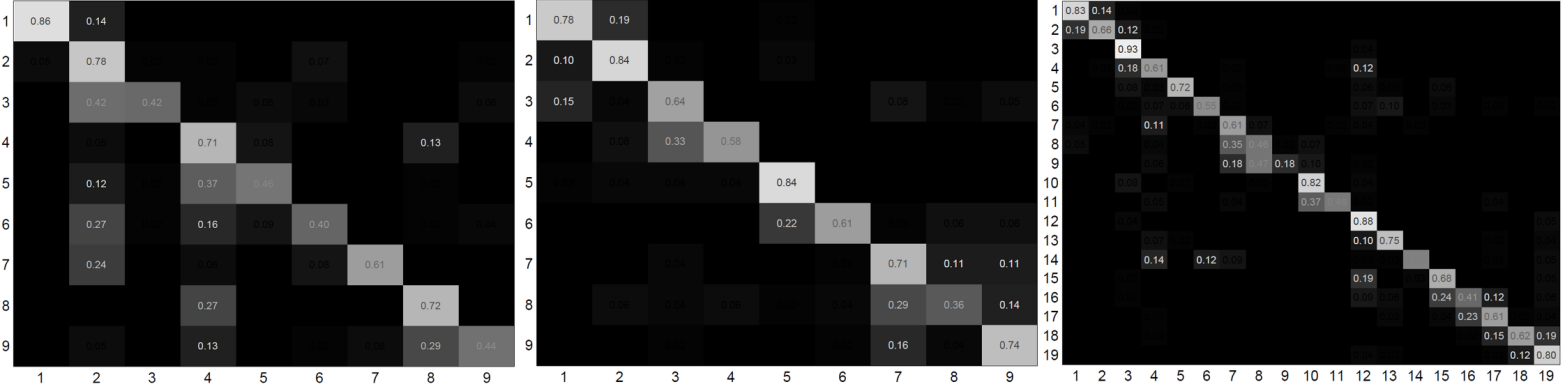
On-line workflow monitoring confusion matrices for *leave-one-subject-out* evaluation. Columns represent the predicted atomic events, while rows represent the actual atomic events. The gray scale value encodes the positive rate from 0 to 1. Left to right: Nails & Screws, Labeling & Packaging, Ball valve.

As a complement to the quantitative results presented above, Fig 14 provides a more global and qualitative view on the performance of the proposed framework and its live activity monitoring capabilities. It illustrates the quality of results obtained using the off-line *leave-one-subject-out* experiment for the Labeling & Packaging dataset (*Object IMU*, column 6 in Table 4). In the figure, vertical lines separate the different workflow executions of each subject. Each atomic event is assigned a unique color and the bottom bar of each subject shows the ground truth. At a given instant the prediction is correct, if the colors of both bars are identical. From the figure it is evident that the prediction often jumps to a wrong atomic event for a short period of time, *e.g.*, due to misclassifying a known event or an irrelevant action. However, after sufficient information has been observed, the system recovers to the correct event. From the experiments it was also observed that the current atomic event is often confused with the previous and the next event. This is a typical synchronization error for sequential data, which is partly due to the manual assignment of ground truth labels. In fact it is difficult for humans to assign boundaries consistently between consecutive events. The effect can be seen in Fig 15, which shows confusion matrices for the on-line workflow monitoring. In each matrix, the diagonal elements are clearly dominant, which reflects the accuracy of the proposed method. Moreover, the false positives are often either the previous or the next atomic events, which shows the above-mentioned synchronization error. Given the goal of the proposed monitoring system to guide users through workflows while ensuring that each relevant atomic task is properly completed, the reduction of such synchronization errors requires further investigation. A detailed evaluation of this synchronization error is presented in [66].

### 3.2 Technical Evaluation of Low-level Sensor Processing

As indicated above, the performance of workflow recovery and monitoring depends on the performances of the underlying low-level sensor processing components; *e.g.*, on consistent and repeatable results independently of the user and the environment. Moreover, in the context of real-time user assistance, the scalability of the processing algorithms with respect to increasing workflow complexity (in terms of number of involved objects) is an important aspect. Hence, this section provides evaluation results of user and scene monitoring concerning the above mentioned requirements.

#### User Monitoring

When capturing the first test dataset, Nails & Screws, it became apparent that severe magnetic disturbances result in inconsistent motion capturing results when purely relying on magnetometer based aiding. This leads to deteriorating workflow recovery and monitoring performance. This was one major motivation for developing the proposed method based on egocentric visual aiding. Consequently, the evaluation of the proposed visual-inertial motion capturing system focuses on its ability to provide consistent and repeatable results independently of magnetic disturbances induced by the environment or tools that the user interacts with. Therefore, rather than validating the proposed approach in terms of absolute precision, a comparative analysis with respect to a commonly used magnetometer based system is provided in the following.

**Fig 16:**
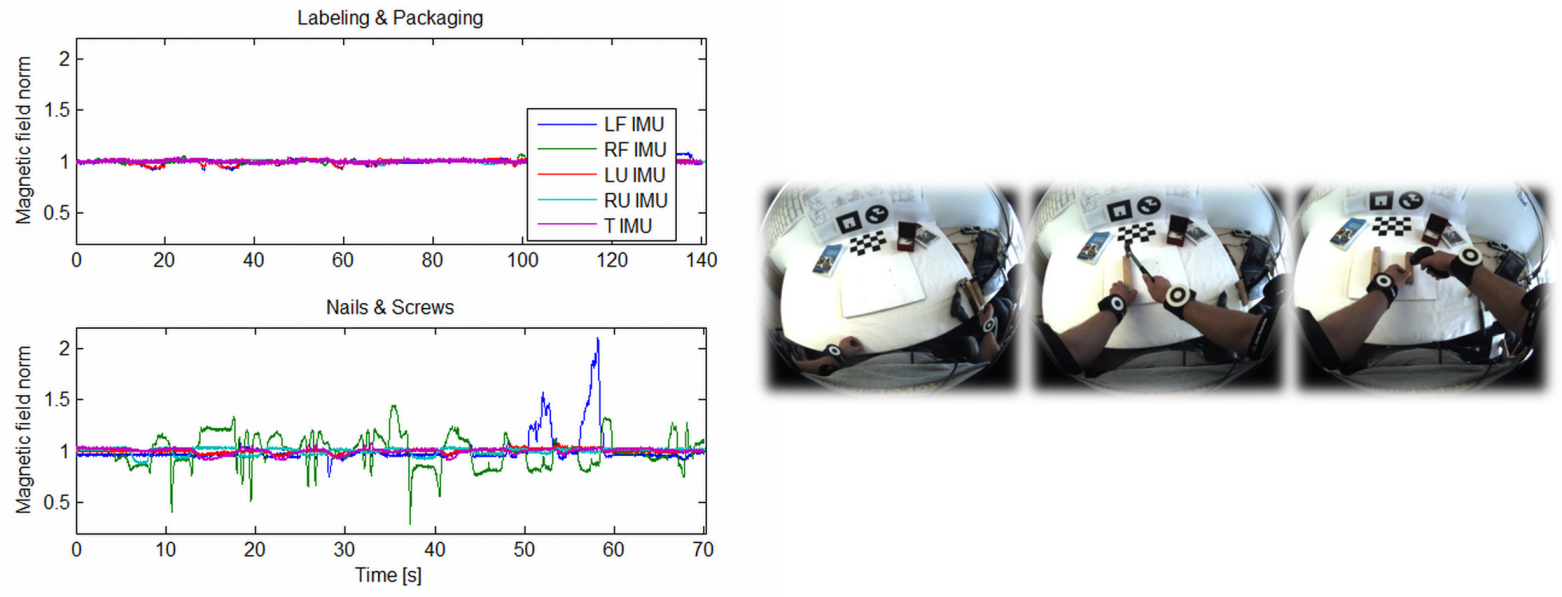
Magnetic disturbances. The plots illustrate the magnetic disturbances occurring during the Labeling & Packaging and the Nails & Screws workflow. In a disturbance-free environment, the norm stays constant under arbitrary poses and motions. While the changes are moderate for Labeling & Packaging, significant changes are noticeable for Nails & Screws, especially in the forearm IMUs (RF IMU, LF IMU). This happens primarily in situations, where the hands approach or handle the hammer or electrical screwdriver. The three chest camera frames correspond to the peak disturbances at second 11, 20, and 52 of Nails & Screws.

The evaluation method consists in comparing the motion capturing results (in terms of estimated 3D wrist positions in the chest camera frame and deviations observed in the images of the chest camera) under different system configurations (magnetometers *vs.* visual wrist detections) and in different situations (absence (Labeling & Packaging workflow) and presence of magnetic disturbances (Nails & Screws workflow)). As illustrated in Fig 16, the Nails & Screws dataset contains severe disturbances introduced by approaching or handling the hammer made of iron or the electrical screwdriver.

**Fig 17:**
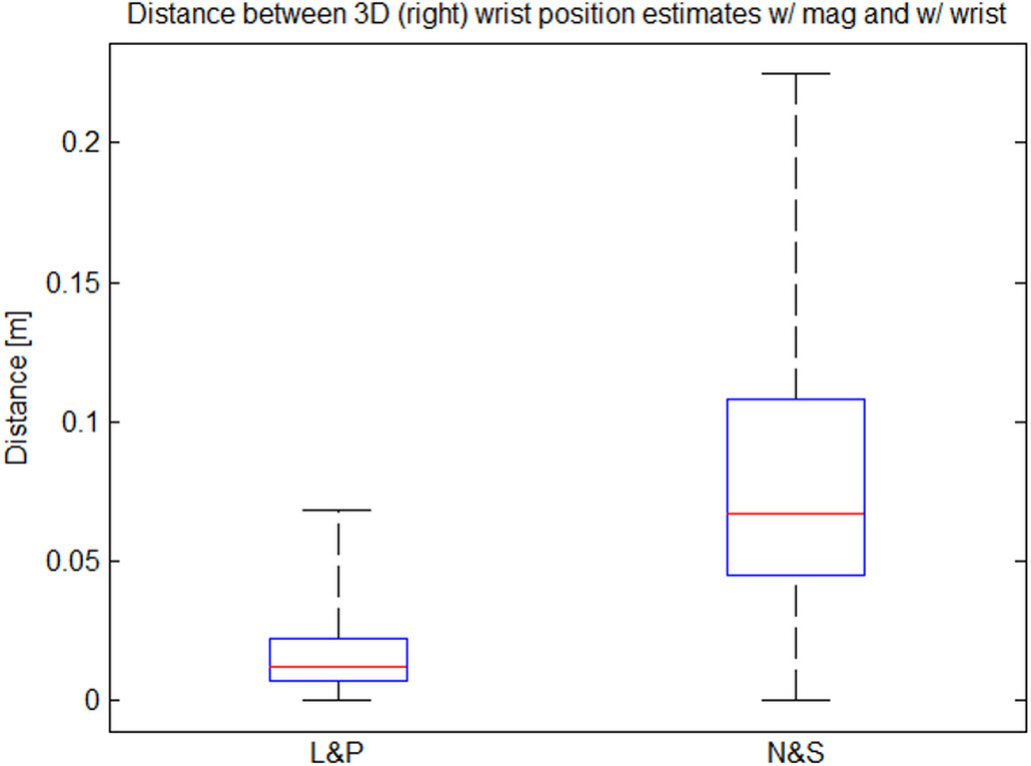
Effects of magnetic disturbances. The plot illustrates the deviation between the 3D wrist position estimates resulting from the magnetometer based and the wrist detection based approach. The data corresponds to the right wrist position estimated in a disturbance-free environment (Labeling & Packaging) and in a disturbed environment (Nails & Screws) for exemplary data sequences.

Fig 17 presents the results when applying the two different system configurations, using magnetometers and using wrist detections, to exemplary data sequences from the two workflow datasets. The estimates obtained from the two configurations deviate significantly from each other in the presence of magnetic disturbances, while a good correspondence is shown, when no disturbances are present.

**Fig 18:**
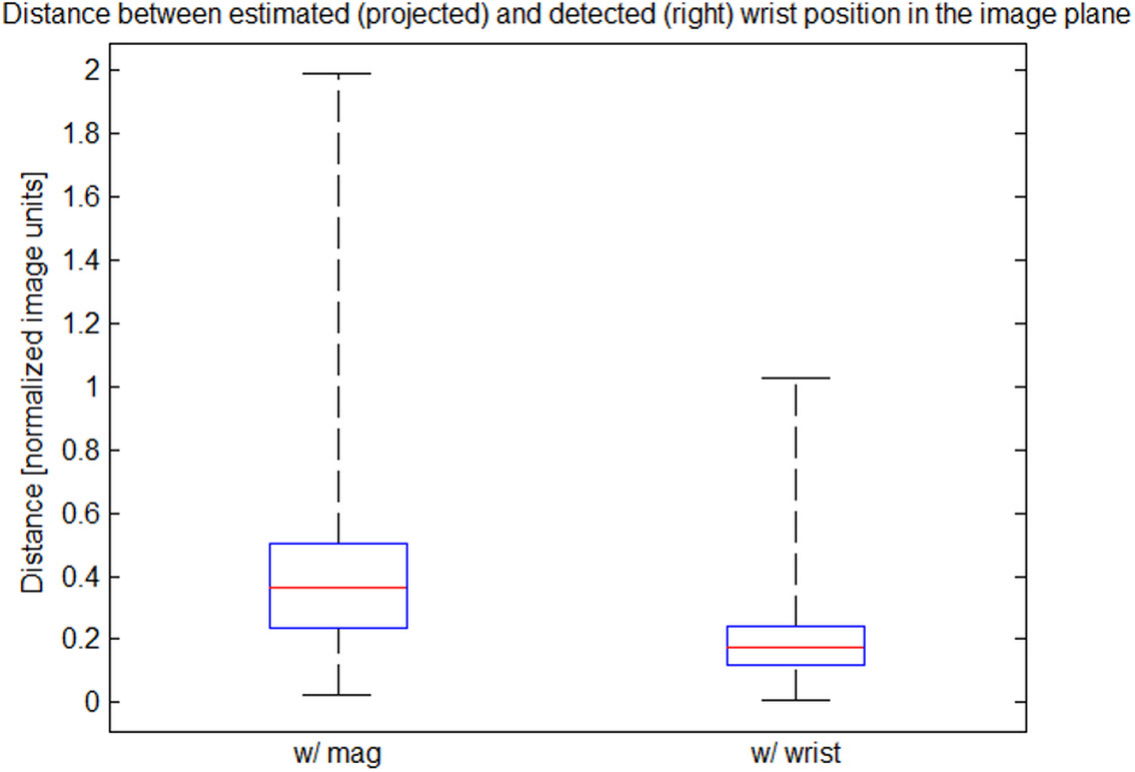
Effects of magnetic disturbances in the image plane. The plot shows the statistics of the deviations between the projected and the detected wrist positions resulting from the magnetometer and the wrist detection based approach. The data corresponds to the right wrist position estimated in an exemplary data sequence of the Nails & Screws workflow. Clearly, the wrist based method outperforms the magnetometer based method.

**Fig 19:**
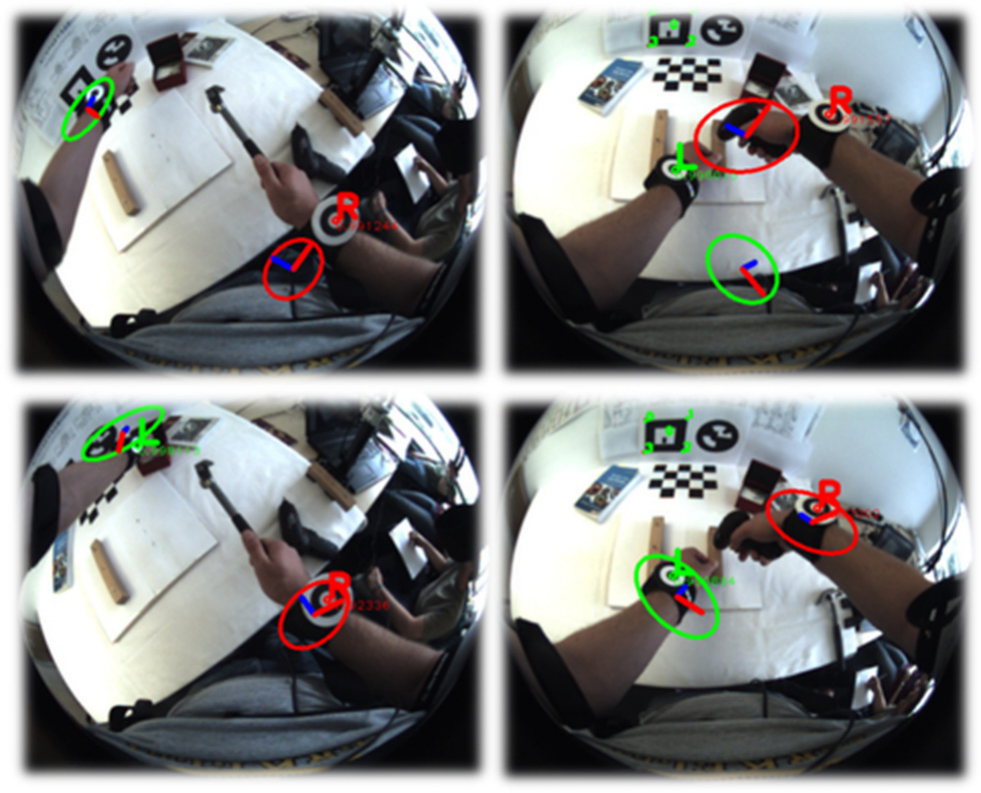
Effects of magnetic disturbances in the image plane. The picture shows example views from the chest camera augmented with the projected wrist position estimates (ellipses) resulting from the magnetometer based (upper row) and the wrist detection based (lower row) approach during disturbances by handling the hammer and the electrical screwdriver. The ellipses indicate the projected uncertainties. The magnetometer based approach results in clear deviations, while the wrist detection based approach shows good matches.

Moreover, the residual errors in the image plane as illustrated in Figs 18 and 19 for Nails & Screws prove the better performance for the wrist detection based method. These results show that the magnetic disturbances in the tested industrial setting are severe enough to deteriorate the magnetometer based solution by propagating the disturbances into the estimated pose and that the proposed egocentric visual-inertial approach outperforms the magnetometer based system in such situations, while otherwise providing a comparable performance. Hence, the proposed method is able to provide consistent input data to workflow recovery and monitoring, independently of magnetic disturbances, which are a typical problem in industrial environments.

#### Workspace Monitoring

As mentioned in Section 3.1, the performance of workflow recovery and monitoring depends on accurate object identification and tracking input. Hence, this section reports respective evaluation results for the three test datasets. Moreover, the scalability of the object detection approach as well as the importance of the per person learning-based approach for achieving person independence on workflow monitoring level are evaluated.

**Fig 20:**
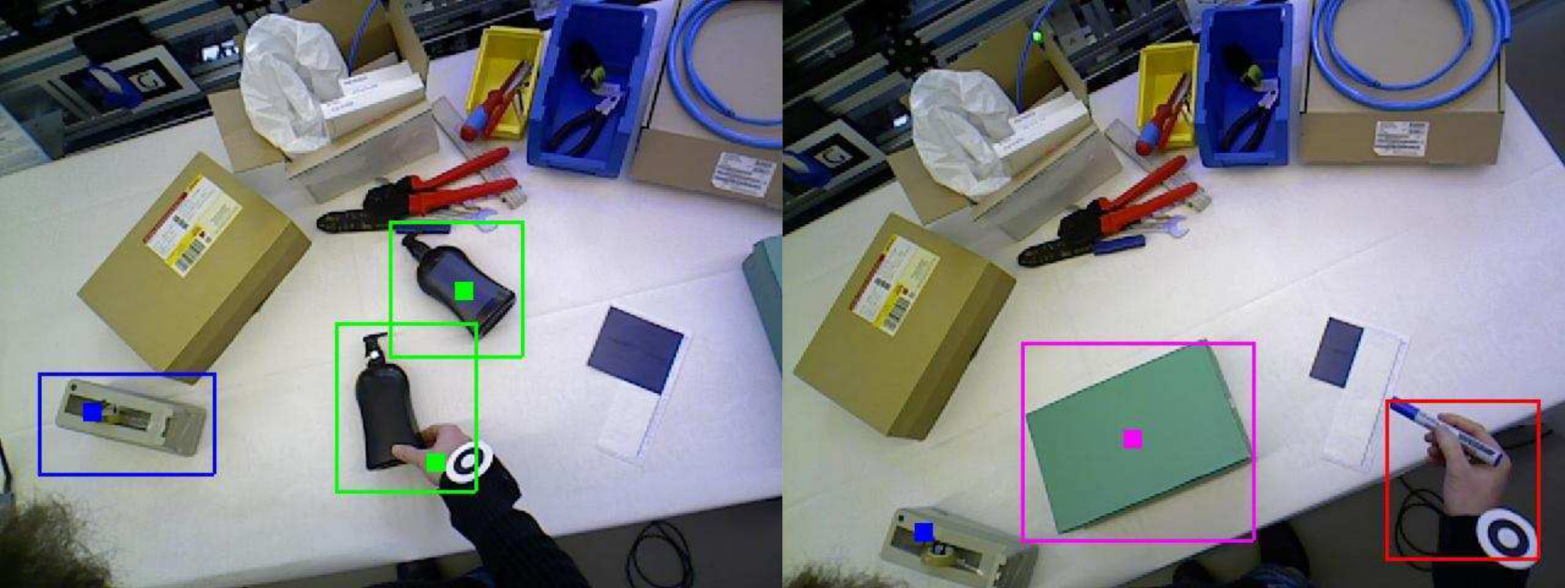
Object recognition and tracking for Labeling & Packaging. Two frames showing ground truth bounding boxes and corresponding output of the method projected onto 2D images for the Labeling & Packaging dataset. Matching colors indicate correct identities. In the bottom right, the tape dispenser was not ground truth labeled (false-positive), and the pen not recognized resulting in a false negative.

For object recognition and tracking, one sequence from each operator was randomly chosen from the Labeling & Packaging task, and manually ground truth labeled with bounding boxes reflecting the different objects present in each frame along the sequence. Recall that the output of the method described in this paper is the 3D trajectory of the object. In comparing to the ground truth, the 3D positions were projected back to 2D given the camera’s position and viewpoint. A true positive occurs when the position of the object falls within the ground truth bounding box. Fig 20 shows examples of comparing to the ground truth. The two common information retrieval measures, precision and recall, were used to assess object recognition and tracking.

**Fig 21:**
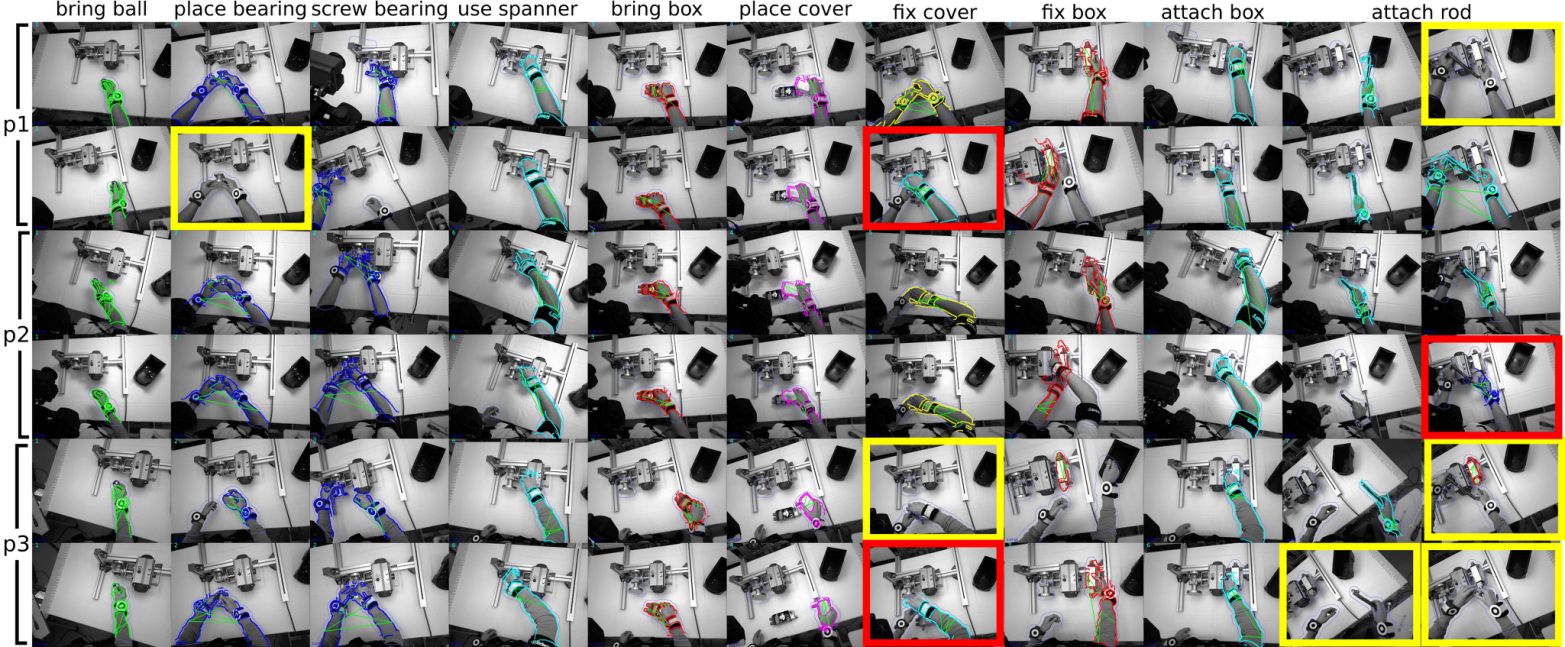
Object recognition and tracking for Ball valve. Frames showing the Ball valve task for 6 sequences from 3 different operators across the task’s primitive events. False negative frames (yellow-bounded) indicate cases where the object failed to be recognized due to a significantly different grasp between learning and testing, or due to occlusion. False positive cases (red-bounded) are due to ambiguous grasps of objects, particularly the spanner and the screw driver, when seen from an overhead camera.

Fig 21 shows sequences of frames from multiple operators for the Ball valve task along with some recognition results. Table 5 presents quantitative results for task-relevant object tracking on the Labeling & Packaging task. The failure to recognize objects is usually related to the object being mostly occluded during the task performance. Another limitation is the usage of cluster-based tracking. The tracker currently maintains a single identity for each cluster. For example, when the pen is writing onto the box, only one cluster is tracked. The pen’s identity is thus often ignored by the tracker, though it achieves good recognition results. Another source of failure shown particularly in Fig 21 is due to ambiguous grasps for small objects (for example the hand-held plier and the hand-held spanner), or the fact that the grasp during learning differed from the grasp while performing the task. This could be improved by adding more discriminative features for similar objects, and by learning more views of the objects during task performance. These improvements are left for future work.

**Table 5:** Object recognition and tracking results for the Labeling & Packaging task. The average precision and recall are shown from 2 runs of each worker.

	**# of frames**	**# of ground truth objects**	**recall**		**precision**	
			*μ* [%]	*σ* [%]	*μ* [%]	*σ* [%]
**subject 1**	4635	12793	57.72	0.03	88.67	0.02
**subject 2**	2768	5631	79.53	0.04	77.36	0.04
**subject 3**	3807	8730	66.81	0.03	90.70	0.02
**subject 4**	3128	5523	75.99	0.05	77.15	0.05
**subject 5**	3259	6613	66.50	0.01	66.82	0.01
**avg**			69.31		80.14	

**Fig 22:**
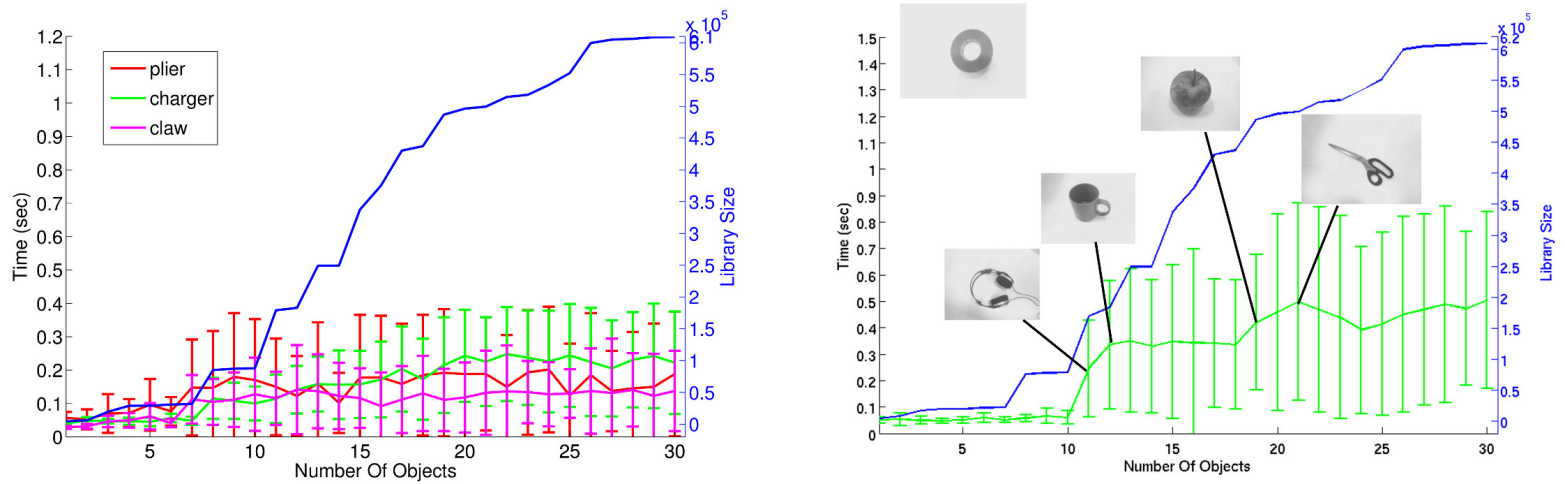
Object recognition results. As the number of objects increases from 1 to 30, the library size increases by more than 150×, while the average detection time increases by 3.3× (plier), 4.8× (claw) and 5.5× (charger). For the object ‘tape’ (right), the average detection time increases by 10×, particularly when objects with a circular shape are learnt (headphone, mug, apple, and scissors).

Further results are presented in [58]. In particular, [58] contains a comparison to state-of-the-art shape-based object detectors in an off-line mode on the standard ETHZ dataset [88], where the proposed approach achieves competitive performance. However, compared to state-of-the-art approaches, the proposed method is distinct in that it can learn and recognize multiple shape-based objects in real-time. Fig 22 plots the detection time as more objects are learnt. The detection time is the elapsed time until the object is correctly detected. The increase in detection time results from comparing to a larger number of descriptors in the hashtable as well as assessing ambiguous matches. From the figure, adding unambiguous objects does not affect the average detection time much. For the ambiguous object ‘tape’ (*cf.* Fig 22 right), the average detection time increases by 10 folds when 30 objects are being searched for. Alternative real-time shape-based object detectors [89, 90] scale linearly with the number of objects. In summary, the above mentioned results show a compatible recognition performance of the proposed approach while at the same time providing superior scalability when it comes to workflows involving a higher number of objects.

**Fig 23:**
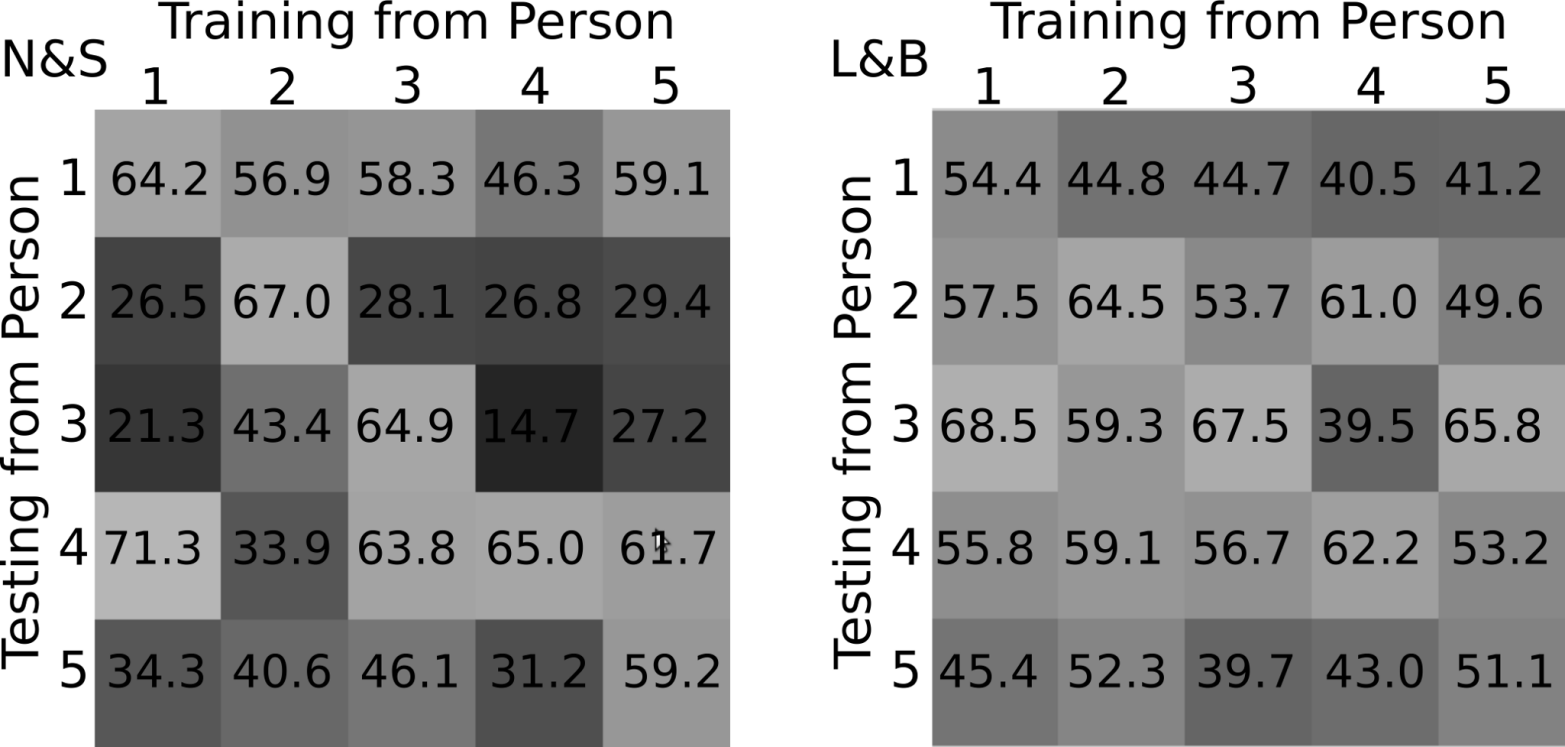
Object recognition results. Confusion matrix showing the accuracy when objects are learnt from a different operator for the Nails & Screws (left) and the Labeling & Packaging (right) tasks.

To show the importance of real-time learning of personal grips, the distinctiveness of the descriptors was evaluated by learning from the manipulation sequence of one operator then testing on other operators. The results are presented in a confusion matrix (see Fig 23). Each cell in the matrix measures the ability to recognize hand-held tools in sequences of operator (row) when learning is done from the grips of another operator (column). For the second operator in the Nails & Screws task, for example, the accuracy drops from 67.0% to a maximum of 29.4% when a different individual’s manipulation sequences were used for learning object grips. For the the Labeling & Packaging task, the maximum drop was from 67.5% to 39.5% for the third operator. These results highlight the importance of the learning-based approach for object grips, as each user has a different way of manipulating tools which affects the visual recognition of objects. Learning person-specific object manipulations is online and is required once per operator. Once the objects are learnt, the operator can use these objects for multiple workflows. While the low-level learning of objects is person-dependent, the workflow monitoring (*cf.* Section 3.1) does not need to be adapted or changed for new operators. We thus believe that introducing this learning-based approach for person-specific object manipulations enables learning and recognition of workflows from individuals with varied grips as well as different tool shapes and sizes. It should be noted that the system assumes the operator knows how to grip the tools and does not need guidance on handling tools. We find this compromise acceptable, as it is the workflow that we wish to monitor rather than fine-grained object gripping.

### 3.3 User Interface Evaluation

In addition to the technical evaluation results presented above, this section presents a qualitative summative evaluation of the final user interface during online workflow assistance, in particular, aiming at conclusive statements on the quality, *i.e.*, the strengths and weaknesses of the final AR player. Recall that accompanying studies throughout the development process both involving potential naive users and usability experts resulted in the final design of the user interface (*cf.* Section 2.5 and [48, 83]). According to a formative evaluation approach, those iterative cross-validations are necessary for a user-friendly output.

#### Evaluation Methods

A final heuristic evaluation was performed by again asking four male usability experts from the German Research Center for Artificial Intelligence, who are working in the fields of AR, usability, and human-machine interaction. Two of these persons had no previous experience from the prototype or the Ball valve workflow; the other two knew the system and the workflow through participation in the previous usability evaluation (*cf.* Section 2.5) and they were able to make qualitative statements on the incremental development from the initial to the final version. None of the recruited usability experts were, however, involved in the system development or the technical evaluations of workflow recovery and monitoring (*cf.* Section 3.1) and they performed the evaluation independently. The explicit role of the experts was to evaluate the user interface based on their knowledge of usability and AR applications. The goal was to explore how they experienced the changes compared to the former version. First, each expert used the prototype and the Ball valve scenario to become familiar with the user interface. Then, by help of a structured interview, which was based on a paper guide containing established usability principles [81], the interviewer encouraged each expert to report as many thoughts as possible on certain aspects and on the user interface as a whole. The aim was not to evaluate the performance of the expert in the scenario, but to obtain subjective opinions about the appearance and performance of the user interface. The whole procedure took around 45 minutes per participant, including detailed familiarization with the system, reflecting on the user interface’s performance, and answering the open usability questions.

#### Results and Discussion

All assessed statements were grouped according to relevant aspects of the user interface. The results of the qualitative data analysis are reported and discussed in the following.

#### 3D animated graphical overlays

The regions of interest and animated arrows displaying local and directional information were comprehensible and supportive according to all experts. Yet, they noted their limitations when it comes to more complex steps in which the exact way of assembly is important. In these cases, they suggest to incorporate more advanced arrows or more complex animations, like video clips (see below) of the object assembly.

#### Video clips on demand

All experts rated the short video clips as very helpful in cases where users require more detailed information during execution of an assembly operation. Video clips immediately transmit the necessary and complete information while inferring little cognitive load. Yet, the attentional focus on the actual workspace and the task to be done is distracted by displaying the video.

#### Textual instructions

The readability of the text was rated as sufficient. The experts also did not report any problems with the visibility of the system status rendered by displaying the current and overall steps. Taking into account the space restrictions of an HMD which does not enable an illustrative workflow overview, the current solution meets the minimum requirement of communicating structural information in step-by-step instructions [72].

#### Audio output

The expert evaluation confirmed that the extended, verbal information via audio adds value to the user interface, in addition to the short, visually displayed, textual instructions. This goes along with the cognitive theory of multimedia learning [71] which advises to design instructional material that targets both sensory channels, auditive and visual. In the study, experts rated audio output as less favorable compared to augmented objects and video clips, but noted that it might be helpful in implicitly and concomitantly spreading the relevant keywords and information. Current work confirms that verbal guidance facilitates cognitive processes during manual tasks [91].

#### Multimodality

Experts did not agree on which modality (augmented graphical overlays, audio output, text, or video clips) or which combination of them to prefer over the others. This goes along with evidence for interindividual differences in preference for media [92]. Consequently, it seems reasonable to offer the selection of modalities previously during authoring (*cf.* Section 2.5). In general, the usage of both graphical and verbal media is beneficial, since their respective advantages and disadvantages can be balanced by combining them [93].

#### Speech input

According to the experts’ statements, additional interaction possibilities via speech commands, where the user can, *e.g.*, query more information, meets the system requirement to provide user control and freedom [81]. In the opinion of the experts, the commands were easy to remember but could nevertheless be shortened in order to facilitate the user’s control and memory processes. At the same time, technical restrictions prevented the usage of shorter commands, since the recognition likelihood decreases.

#### Attentional guidance

The arrows in the corners or margins of the screen were evaluated as intuitively understandable and appropriate to guide the user’s attention to the relevant location on the workspace.

In sum, no usability expert reported major violations to usability principles in the presented user interface. The user interface, furthermore, provides a choice of several options that meet individual preferences, specific task characteristics, and current evidence on multimedia instruction.

## 4 Conclusion

As the tasks needed to be performed in various industrial situations (*e.g.*, assembly or repair), become increasingly complex, the importance of assistive technology is obvious. The prototype system presented in this article represents one such assistance system. To achieve maximal flexibility, it requires no external sensors or infrastructure for proper operation. To reach this state, advances in hardware, software, and at system level have been necessary to close the action-perception-feedback loop in the demonstrator. Considerable advances are described and evaluated for all the comprised components: user pose estimation, positioning important objects relative to the workspace, workflow recovery and monitoring, and presenting information to the user.

For the user monitoring, a method to derive the user pose based on measurements from IMUs and a camera has been presented and shown to solve the problem also in presence of magnetic disturbances. This is in contrast to similar existing egocentric systems, which cannot handle magnetic disturbances. Ongoing work is aiming to remove the need to put markers on the user’s wrists by integrating a marker-less hand detection and tracking approach [94–96], which in combination with even more miniaturized sensors (*e.g.*, [82]) would make the system truly unobtrusive.

For the workspace analysis, a learning-based method to detect and track objects with minimal texture and under manipulation has been outlined. This is achieved by using the relative orientation of the object’s edges (edgelets) to provide a scale and rotation invariant feature to detect and by modelling the user’s grip together with the object. The method scales gracefully with the number of objects in the scene and object database. It distinguishes from state-of-the-art methods in that it can learn and recognize multiple shape-based objects in real-time. To help in the segmentation task and position the objects in 3D, the background scene is previously mapped and the sensor is continuously tracked with respect to this using depth information. Future extension of the method includes being able to learn new objects on the fly as well as extending the feature description to better cope with flexible and glossy objects possibly by incorporating color information.

For the workflow recovery and monitoring, a hierarchical workflow model has been presented. A sliding-window based approach is used for live activity recognition. Each window is represented as a bag-of-relations that encodes the frequency of the instantaneous object-object, object-wrist and upper-body parts spatiotemporal relations. The histogram representation of the above bag-of-relations is then used by the proposed model, which is learnt in a supervised fashion by combining an SVM and an HMM. The atomic events are tied to the HMM state. In contrary to the existing approaches, the proposed method is both domain-independent and robust as shown on the actual data from the described system. To further enable easy deployment, methods for unsupervised or semi-supervised learning of the model should be pursued.

For the user interaction and guidance, a multimodal user interface for efficiently guiding users through complex tasks has been developed and evaluated in field studies. The system exploits information from the workflow analysis in order to provide timely instructions in terms of augmented reality (AR) overlays and audio rendered in a head mounted display (HMD). The system could be improved by further investigation of the possibilities provided from using more modalities, *e.g.*, haptic feedback.

All the described parts have been designed with interoperability in mind, allowing for them to be combined into a single demonstrator. This concept has been proven successful, in that the system described in this article has been demonstrated and shown to work on a limited size dataset of increasingly complex industrial tasks. The prototype is designed to be capable of large scale testing in industrial settings, which is indeed a future direction.
